# High-throughput and high-sensitivity full-length single-cell RNA-seq analysis on third-generation sequencing platform

**DOI:** 10.1038/s41421-022-00500-4

**Published:** 2023-01-11

**Authors:** Yuhan Liao, Zhenyu Liu, Yu Zhang, Ping Lu, Lu Wen, Fuchou Tang

**Affiliations:** 1 Biomedical Pioneering Innovation Center, Beijing Advanced Innovation Center for Genomics, School of Life Sciences, Peking University, Beijing, China; 2 Ministry of Education Key Laboratory of Cell Proliferation and Differentiation, Beijing, China; 3 Peking-Tsinghua Center for Life Sciences, Academy for Advanced Interdisciplinary Studies, Peking University, Beijing, China; 4 Changping Laboratory, Beijing, China

**Keywords:** Bioinformatics, Transcriptomics

Dear Editor,

The advancement of single-cell RNA-seq technologies based on third-generation sequencing (TGS) platforms has accelerated biological researches. Several TGS platform-based single-cell RNA-seq methods have been developed since 2016^1^. Limited by low accuracy and sensitivity, they either combine NGS platform-based methods to reduce the error rates^2^, or sacrifice the throughput to improve detection rates^3^. To overcome these drawbacks, here we developed SCAN-seq2, a high-throughput, high-sensitivity full-length single-cell RNA-seq method based on TGS platform. We performed SCAN-seq2 to a total of 5472 cells from nine cell lines. Detailed flowchart of the data processing pipeline and overall statistics of reads after filtering, demultiplexing and deduplication were shown (Supplementary Figs. S1 and S2). Through reference-guided transcriptome assembly, we identified thousands of novel full-length RNA isoforms. Transcripts of pseudogenes could be distinguished from transcripts of corresponding parent genes and hundreds of them showed cell-type-specific expression patterns. Moreover, we showed that V(D)J rearrangement events could be accurately determined for the highly polymorphic T cell receptor (TCR) and B cell receptor (BCR) genes (immunoglobins). Finally, we demonstrated the conserved apoptosis response of HepG2 and Hela cells after treated by spliceosome inhibitor Isoginkgetin (IGG). SCAN-seq2 proves to be a new promising tool for single cell full-length transcriptome research.

In SCAN-seq2, the first-strand cDNAs of every 32 single cells with different 3′ barcodes were pooled together after reverse transcription. By introducing 24-nt barcodes to the PCR primers, different 5′ barcodes were added during PCR amplification. In this way, we were able to sequence up to 3072 (32 × 96) single cells for one sequencing run (Fig. 1a). To evaluate stability and reliability, we tested SCAN-seq2 with different throughput, from 96 to 960 cells, in a total of five groups (Library UMI-100, Library UMI-200, Library 9CL, Library 9CL-mix1~3 and Library 4CL) (Supplementary Table S1) (see Supplementary Method). Taking Library 9CL as an example, the length of cDNA products before library construction was 1500–3000 bp. 75.9% of reads in a sequencing run had complete library structures as designed. The average unique mapping ratio was 83%, which was higher than that of NGS platform-based methods (Supplementary Fig. S3a–e). The number of genes and transcripts detected in each individual cell entered a plateau when the number of reads for each cell exceeded 400,000 (Supplementary Fig. S3g). On average, we detected over 4000 genes and 4500 well-assembled RNA isoforms in each single cell when 960 cells were analyzed for a sequencing run (Fig. 1b). The sequence error counts for each individual 24-bp barcode was less than 3 in general, which was much lower than the edit distance (11 bases) among these 96 different barcodes (Supplementary Fig. S4). We also compared the ERCC UMIs of the same library identified by SCAN-seq2 and NGS-based method. With an edit distance of 1, 86% of the SCAN-seq2 UMIs could be assigned to corresponding NGS UMIs (Supplementary Fig. S5), verifying the reasonable quality of the SCAN-seq2 UMIs. These results showed the combination of high throughput and high sensitivity of SCAN-seq2.

**Fig. 1:**
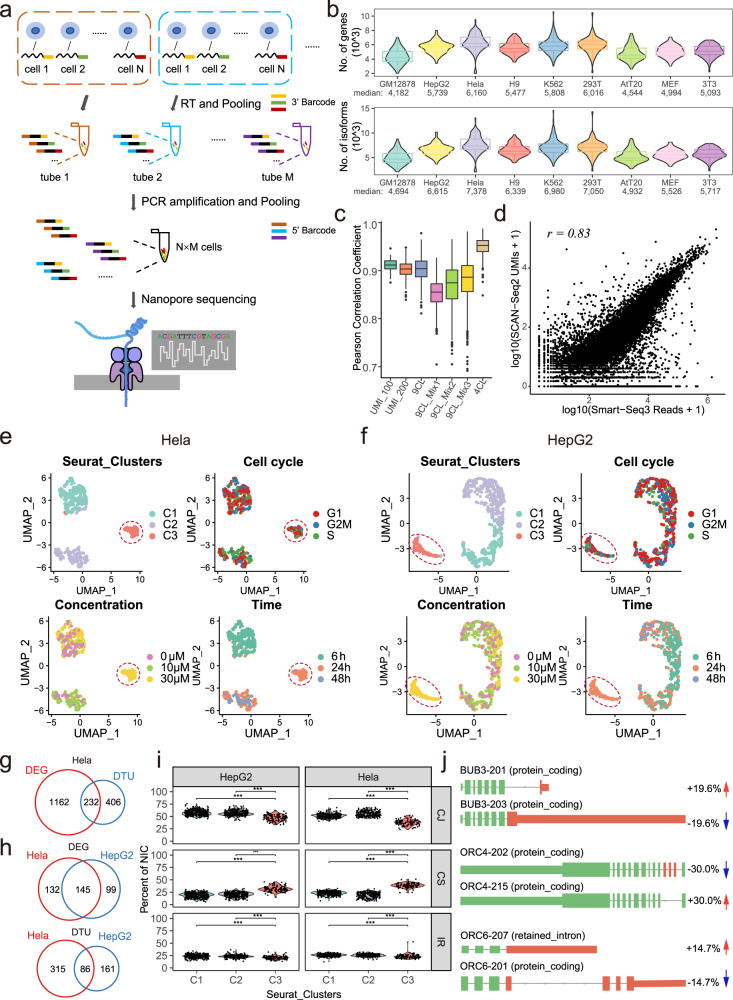
SCAN-seq2 technical performances and analysis of Isoginkgetin (IGG) responses in cancer cell lines. **a** Schematic diagram of SCAN-seq2 library construction. N different single cells are labeled with 3′ barcode during reverse transcription and pooled into the same tube for PCR amplification. M different tubes are pooled together and sequenced with Nanopore platform, allowing parallel sequencing of N×M cells. **b** Number of detected genes (top) and isoforms (bottom) in 852 cells from library 9CL. Median values are labeled under each cell line. **c** Pearson correlation of ERCC concentration and sequenced UMIs in each library. **d** Correlation between gene expression quantification of SCAN-seq2 and Smart-seq3 in 293 T cells. Single cells are aggregated into pseudo bulk for comparison. **e**, **f** UMAP embeddings of Hela (**e**) and HepG2 (**f**) cells after IGG treatment at different concentration and time. Cells are colored by unsupervised clustering results (top left), cell cycle phase (top right), IGG concentration (bottom left) and time of treatment (bottom right). IGG-responsive clusters are highlighted in red circles. **g** Venn diagram showing the overlap in upregulated differentially expressed genes (DEGs) and differential transcript usage (DTU) in IGG responsive cluster of Hela and HepG2 cells. **h** Venn diagram of DEGs and genes with significant DTU in IGG-responsive cluster of Hela cells. **i** Fraction of each subcategory for NIC transcripts in different clusters. *P* values are calculated by two-tailed Wilcoxon rank-sum test. ***P < 0.001. **j** Examples of genes with significant differential transcript usage in IGG responsive cluster. Exons with different usage are highlighted in red.

We then assessed the level of cross contamination. After stringent quality control, essentially reads of all cells from Library 4CL were aligned to mouse or human reference transcriptome correctly, and only one cell (0.28%) was identified as contaminated, confirming the low cross-contamination rate of SCAN-seq2 (Supplementary Fig. S3f). With UMIs added, SCAN-seq2 achieved comparable accuracy to the golden standard method—Smart-seq3. The Pearson correlation coefficient between UMI counts and actual ERCC concentration was higher than 0.85 in all 5 libraries (Fig. 1c). The correlation on global gene expressions between merged data of SCAN-seq2 and Smart-seq3 was 0.83, verifying the consistency between these two methods (Fig. 1d). Additionally, the average correlation between each pair of individual cells reached 0.95, which was as high as Smart-seq3 (Supplementary Fig. S3h), suggesting great reproducibility of SCAN-seq2.

Unsupervised clustering analysis of Library 9CL identified 9 clusters, corresponding exactly to 9 known cell lines. Expressions of well-known marker genes further confirmed the identity of each cluster (Supplementary Fig. S6). In particular, SCAN-seq2 could unambiguously distinguish different RNA isoforms from the same gene. In our dataset, SCAN-seq2 detected high expression of *PTPRC* (CD45) gene in all 3 human immune cell lines (GM12878, H9, K562). However, the major RNA isoforms of *PTPRC* expressed in them were distinct. GM12878 cells only expressed isoform PTPRC-209, which was translated to protein CD45 RABC. K562 cells only expressed isoform PTPRC-201, which was translated to protein CD45 RO. While isoforms PTPRC-201, PTPRC-203 and PTPRC-209 were simultaneously expressed in H9 cells (Supplementary Fig. S3i, j). CD45 RO, CD45 RBC, CD45 RABC regulated downstream signaling pathways of development to varying degrees. These findings were consistent with previous studies, further verifying the accuracy of SCAN-seq2 to distinguish different RNA isoforms from the same gene at single-cell resolution.

Pseudogenes have been found important in multiple biological processes including development and cancer^4^. SCAN-seq2 could unambiguously distinguish transcripts of pseudogenes from transcripts of corresponding parent genes (Supplementary Table S2). Taking pseudogene *RPS2P46* and corresponding parent gene *RPS2* as an example, there were only 2 bases’ differences in a 400 bp long fragment. A total of 1444 pseudogenes were clearly expressed and on average each human cell line expressed 586 pseudogenes. The expressed pseudogenes were mainly processed and unprocessed pseudogenes, which was consistent with previous study [9]. The highest expression level we identified reached over 500 TPM in an individual cell (Supplementary Fig. S7a–d). We selected 5 highly expressed pseudogenes (TPM > 10) for validation by RT-PCR coupled with Sanger sequencing. All of them were consistent with the results from SCAN-seq2 (Supplementary Fig. S9 and Table S8). Notably, based on the expression patterns of pseudogenes alone, we could separate these six cell lines into different clusters (Supplementary Fig. S8). 270 pseudogenes could be used as an additional set of cell-type specific signature genes (Supplementary Table S2). Furthermore, 884 co-expressed pseudogene-parent gene pairs were identified, and the expressions of pseudogenes were generally lower than corresponding parent genes, especially for the processed pseudogenes. We identified 9 positively correlated pseudogene-parent gene pairs and 4 negatively correlated pairs, respectively (Supplementary Fig. S7e, f). There might be direct inter-regulations between these pseudogene-parent gene pairs.

We performed reference-guided assembly of single cell transcriptome to reveal unannotated transcripts using Library 9CL, Library 9CL-mix1, and Library 4CL (Supplementary Table S3). All transcripts were classified into 8 known types. Over 80% of them were categorized as either full splice match (FSM) or incomplete splice match (ISM), demonstrating the high quality of the assembly (Supplementary Fig. S10a–c). Over 98% of splicing junctions belonged to known canonical junction type and less than 2% were novel junction type. Unannotated transcripts mainly belonged to novel in catalog (NIC) and novel not in catalog (NNC). We focused on NIC and identified 5140 and 2047 novel transcripts in human and mouse cell lines, respectively. There were three types of NIC, with combination of known junctions (CJ) taking the largest proportion in both species (Supplementary Fig. S10d, e). 14 unannotated transcripts of protein coding genes were selected for validation by RT-PCR coupled with Sanger sequencing, and all were consistent with assembled transcriptome (Supplementary Fig. S11 and Table S8).

SCAN-seq2 could also be used to study TCRs and BCRs, where transcripts are generated after recombination of variable (V), diversity (D), and joining (J) gene segments (Supplementary Figs. S12a and S13a). A large number of antigen receptor-encoding transcripts were detected in both H9 and GM12878. For TCRs, only transcripts of gene *TRAC* and *TRBC1* were detected in H9 cells, indicating the expressions of TCRαβ genes. And we found that essentially all H9 cells had the same V(D)J rearrangement for β chain, with *TRBV13* as the V element, *TRBD263* as the D element and *TRBJ1-2* as the J element. As expected, the D element of α chain was not detected. Two subclones were further identified, corresponding to two VJ rearrangement of α chain (Supplementary Fig. S12b–d and Table S4). The main differences between subclones in BCRs from GM12878 were in the heavy chain, while the light chain was essentially the same (Supplementary Fig. S13b–e and Table S5). The largest GM12878 clone we identified was precisely consistent with the “Ground-Truth Gene Annotations” of GM12878 immunoglobin^5^. These results proved that SCAN-seq2 could accurately determine the transcripts of TCR and BCR genes for both constant regions and V(D)J recombination, permitting accurate detection of the clonal diversity of T cells and B cells at single-cell resolution.

We treated Hela and HepG2 cells with different concentration of IGG, a spliceosome inhibitor, and collected cells at different time points (Supplementary Table S6). Unsupervised clustering analysis after cell cycle regression displayed 3 clusters in both cell lines. We noted that in both cell lines there was a cluster (Cluster 3) separated from other groups, and it was composed of cells treated by 30 μM IGG for 24 h (Fig. 1e, f). This was further confirmed by parallel profiling of the same batches of cultured Hela cells using NGS methods (816 cells) and SCAN-seq2 (783 cells) simultaneously (Supplementary Fig. S14). Differentially expressed gene (DEG) and differential transcript usage (DTU) analysis were performed in Hela cells independently (Supplementary Table S7). We found DTU events in 638 genes between Cluster 3 and the other two clusters. 406 (63.6%) of them were not overlapped with the DEGs, which was difficult to be identified before (Fig. 1g). Remarkably, 70 (17.2%) of these DTU events led to alterations in protein products. In this situation, different RNA isoforms from the same gene were translated to different proteins. And another 27 (6.7%) DTU events led to significant changes in protein coding ability. For these genes, one RNA isoform had protein coding potential (could be translated to proteins) while another did not (could not be translated to proteins), potentially altering protein abundance. For example, unlike the high expressions of isoform UPP-203 in Cluster 1 and 2, isoform UPP1-202 was specifically highly expressed in Cluster 3, switching the function of oncogene *UPP1* from protein-coding into nonsense-mediated decay. Although the total RNA level of *UPP1* did not change, the UPP1 protein level was expected to be much lower in Cluster 3. Comparing the DEGs and genes with DTU of Hela cells and HepG2 cells, we found that 145 genes (39%) and 86 transcripts (15%) were specifically expressed in Cluster 3 of both cell lines (Fig. 1h). Gene ontology (GO) analysis of common DEGs of Cluster 3 indicated enrichment of biological terms related to nonsense-mediated decay, including gene *RPS21* and *RPS27*, which were relevant to proliferative and invasive capacities of tumor cells (Supplementary Fig. S15). We also observed that the CS type of unannotated transcripts was most significantly upregulated after IGG treatment in both cell lines (Fig. 1i). Furthermore, cell-cycle related genes *BUB3, ORC4* and *ORC6* were expressed in both cell lines. We found that for all these 3 genes, one isoform was significantly up-regulated and the other was down-regulated after IGG treatment (Fig. 1j). The result showed that SCAN-seq2 could depict the cellular responses to small molecule inhibitors at individual RNA isoform level in single cells, providing further explanation for the cytotoxicity induced by small molecule inhibitors in cancer cells.

SCAN-seq2 allows us to sequence thousands of single cells within one sequencing run. Notably, the cost of an individual cell is reduced to about $3 for a sequencing run of 960 cells, which is 20 times cheaper than SCAN-seq (about $60 for each cell). Additionally, the introduction of UMI ensures the accuracy of transcript quantification. Biological discoveries including pseudogenes, unannotated transcripts and DTU events were validated using orthogonal Sanger sequencing (Supplementary Figs. S9, S11, and S16 and Table S8), which proved the reliability of SCAN-seq2. Compared with other published scRNA-seq methods based on TGS platform, SCAN-seq2 exhibited high throughput and high sensitivity simultaneously (Supplementary Table S9). As a full-length sequencing method, SCAN-seq2 can acquire more uniform transcript information without fragmentation and enrichment. So, to obtain the same coverage for each fragment of the full-length transcripts, the sequencing depth required by SCAN-seq2 can be much lower than short-read sequencing. As a more convenient tool, SCAN-seq2 can be used to study different biological systems at single cell and individual RNA isoform resolution and help understanding the complex mechanisms of many diseases.

## Supplementary information

Supplementary Figures and Methods

Supplementary Table S1. Summary of sequenced cells

Supplementary Table S2. Systematic evaluation of pseudogen expression

Supplementary Table S3. Classification of transcriptome assembly

Supplementary Table S4. V(D)J recombination of TCR

Supplementary Table S5. V(D)J recombination of Immunoglobulin

Supplementary Table S6. Summary of IGG-treated cells

Supplementary Table S7. DEG and DTU for IGG-responsive cluster

Supplementary Table S8. Validation of key findings

Supplementary Table S9. Comparison of SCAN-seq2 and other long-reads scRNA-seq methods

## Data Availability

All relevant data are available from the Gene Expression Omnibus (GEO) database (accession number: GSE203561).
