# Publisher Correction: High-throughput and high-sensitivity full-length single-cell RNA-seq analysis on third-generation sequencing platform

**DOI:** 10.1038/s41421-023-00522-6

**Published:** 2023-02-07

**Authors:** Yuhan Liao, Zhenyu Liu, Yu Zhang, Ping Lu, Lu Wen, Fuchou Tang

**Affiliations:** 1 Biomedical Pioneering Innovation Center, Beijing Advanced Innovation Center for Genomics, School of Life Sciences, Peking University, Beijing, China; 2 Ministry of Education Key Laboratory of Cell Proliferation and Differentiation, Beijing, China; 3 Peking-Tsinghua Center for Life Sciences, Academy for Advanced Interdisciplinary Studies, Peking University, Beijing, China; 4 Changping Laboratory, Beijing, China

**Keywords:** Bioinformatics, Transcriptomics

Correction to: *Cell Discovery* (2023) 9:5

10.1038/s41421-022-00500-4 published online 11 January 2023

During the production, we mislabeled all the authors as corresponding authors. The corrected one appeared as below. We apologize for any inconvenience that it may have caused.

Correspondence: Fuchou Tang (tangfuchou@pku.edu.cn)

