# Supplementary material for: High-throughput and high-sensitivity full-length single-cell RNA-seq analysis on third-generation sequencing platform
Source: Cell Discov. 2023 Jan 11;9:5. doi: 10.1038/s41421-022-00500-4 (PMC9834245; doi:10.1038/s41421-022-00500-4)
Supplement: Supplementary file 1 — Supplementary Figures and Methods [file 41421_2022_500_MOESM1_ESM.pdf]

Supplementary Fig. S1

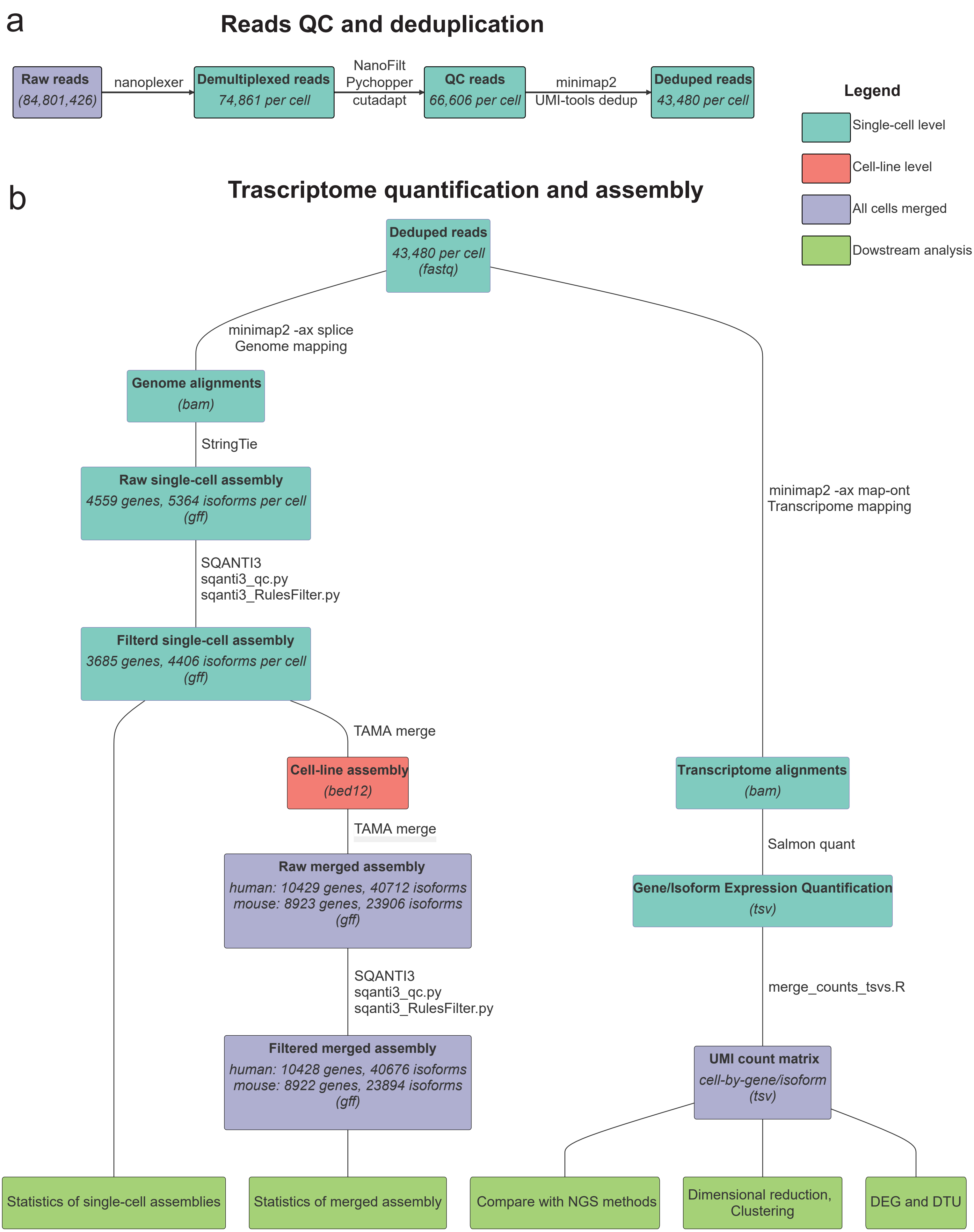

Supplementary Fig. S2

a

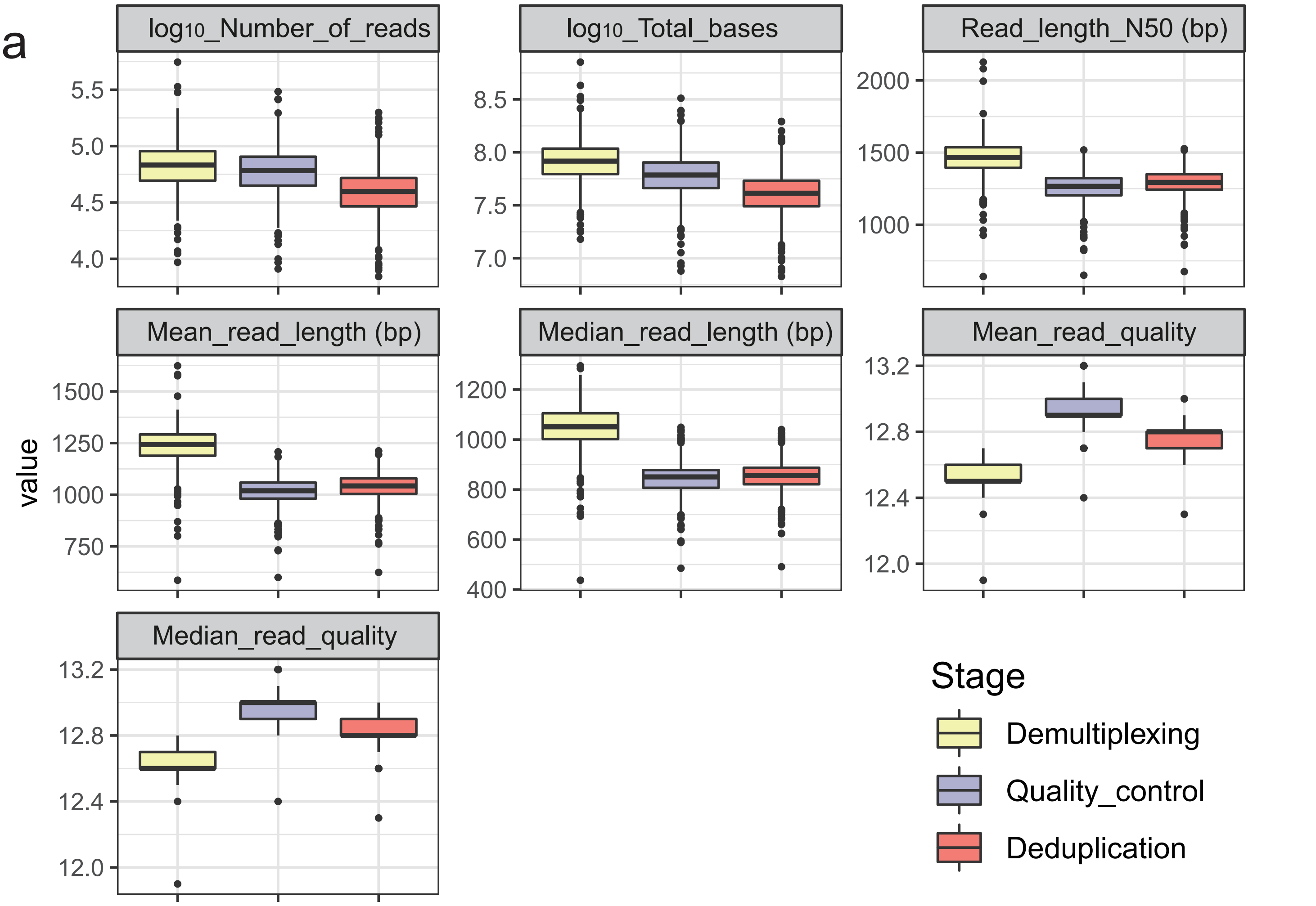

b

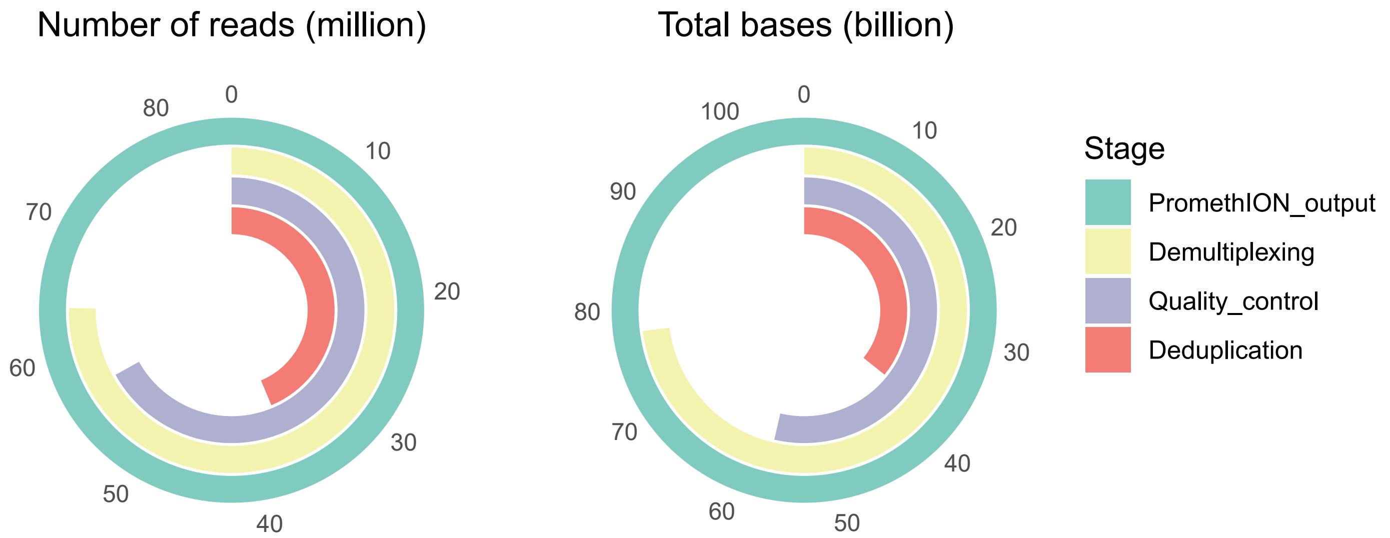

Supplementary Fig. S3

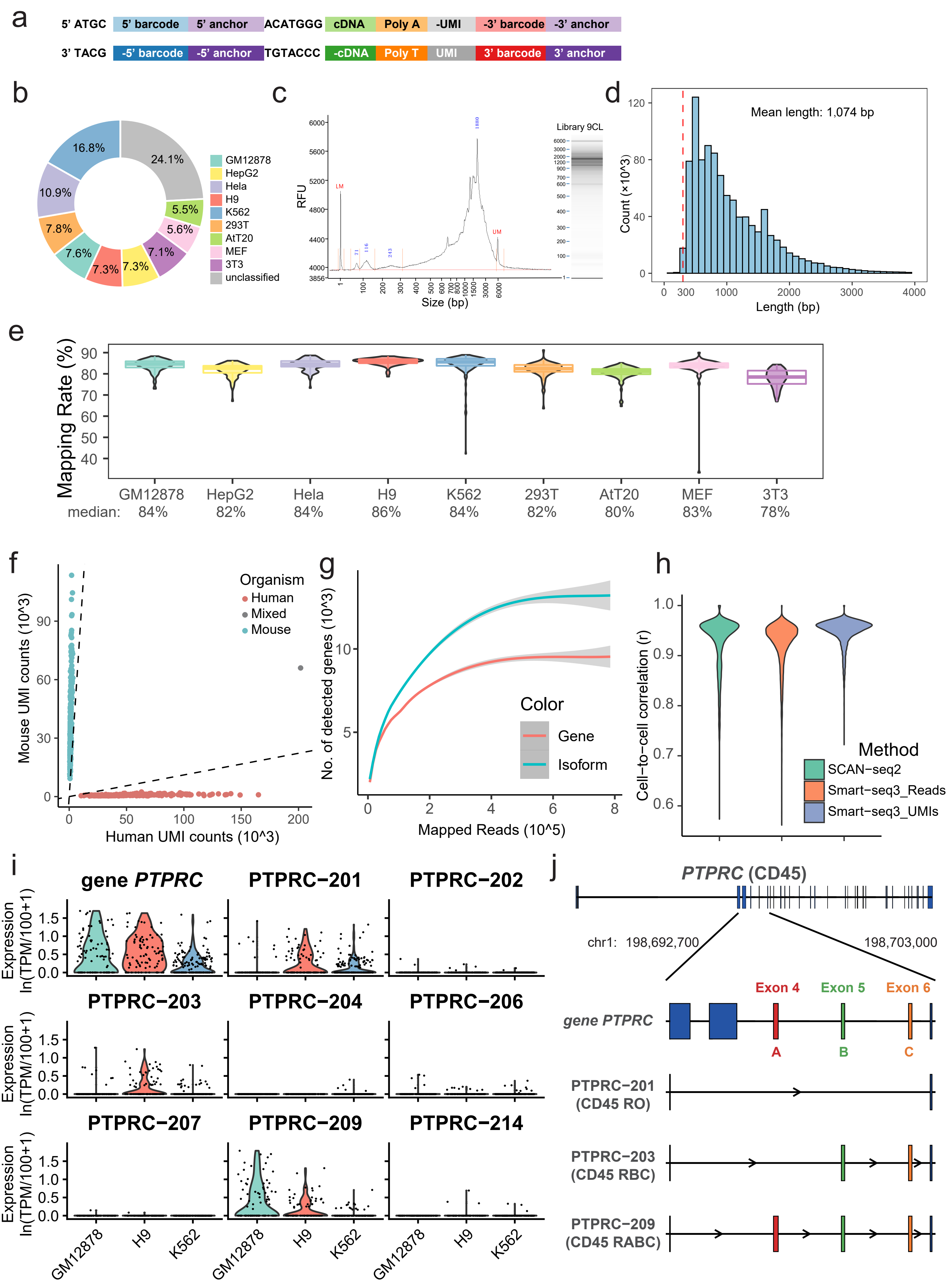

Supplementary Fig. S4

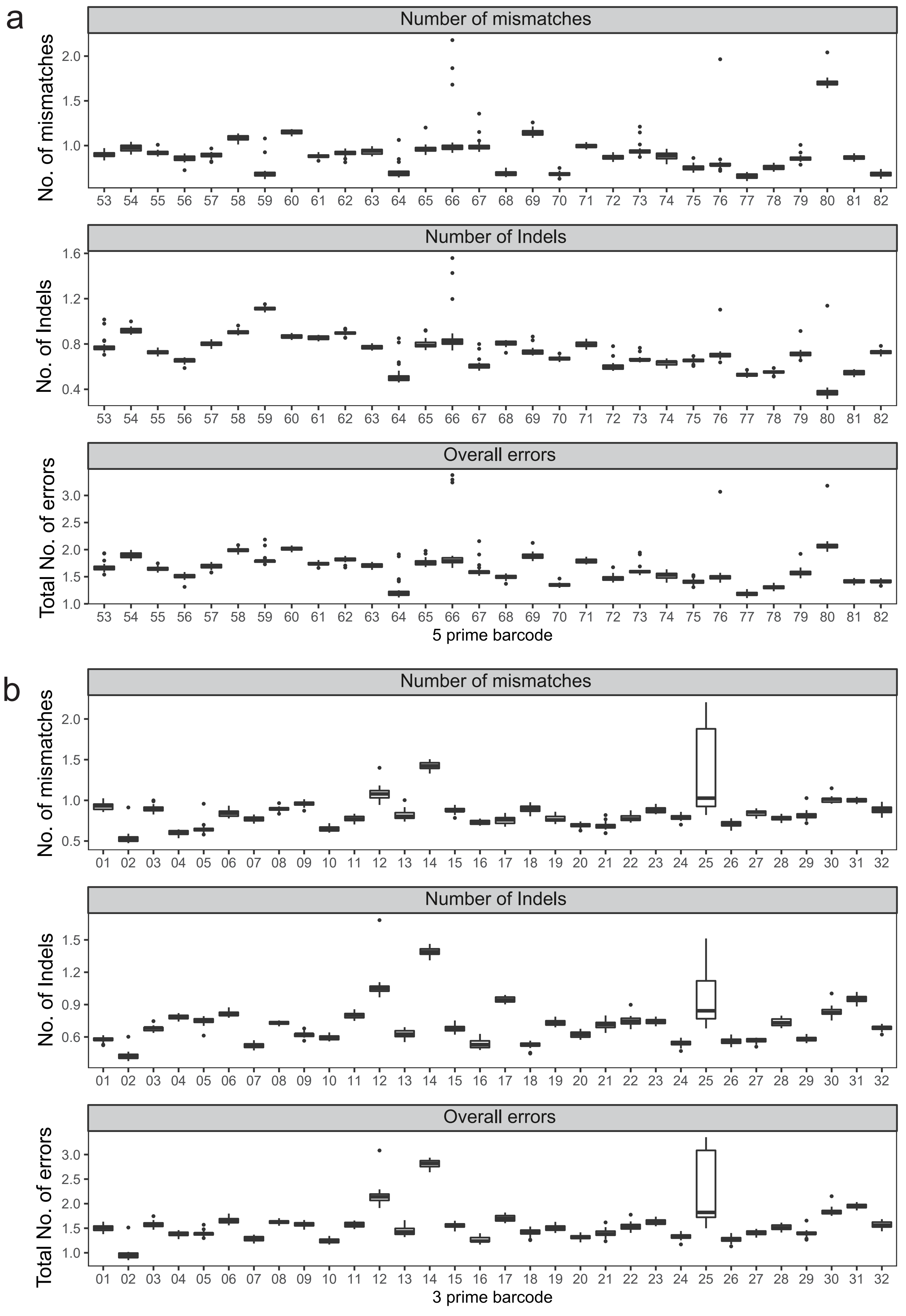

Supplementary Fig. S5

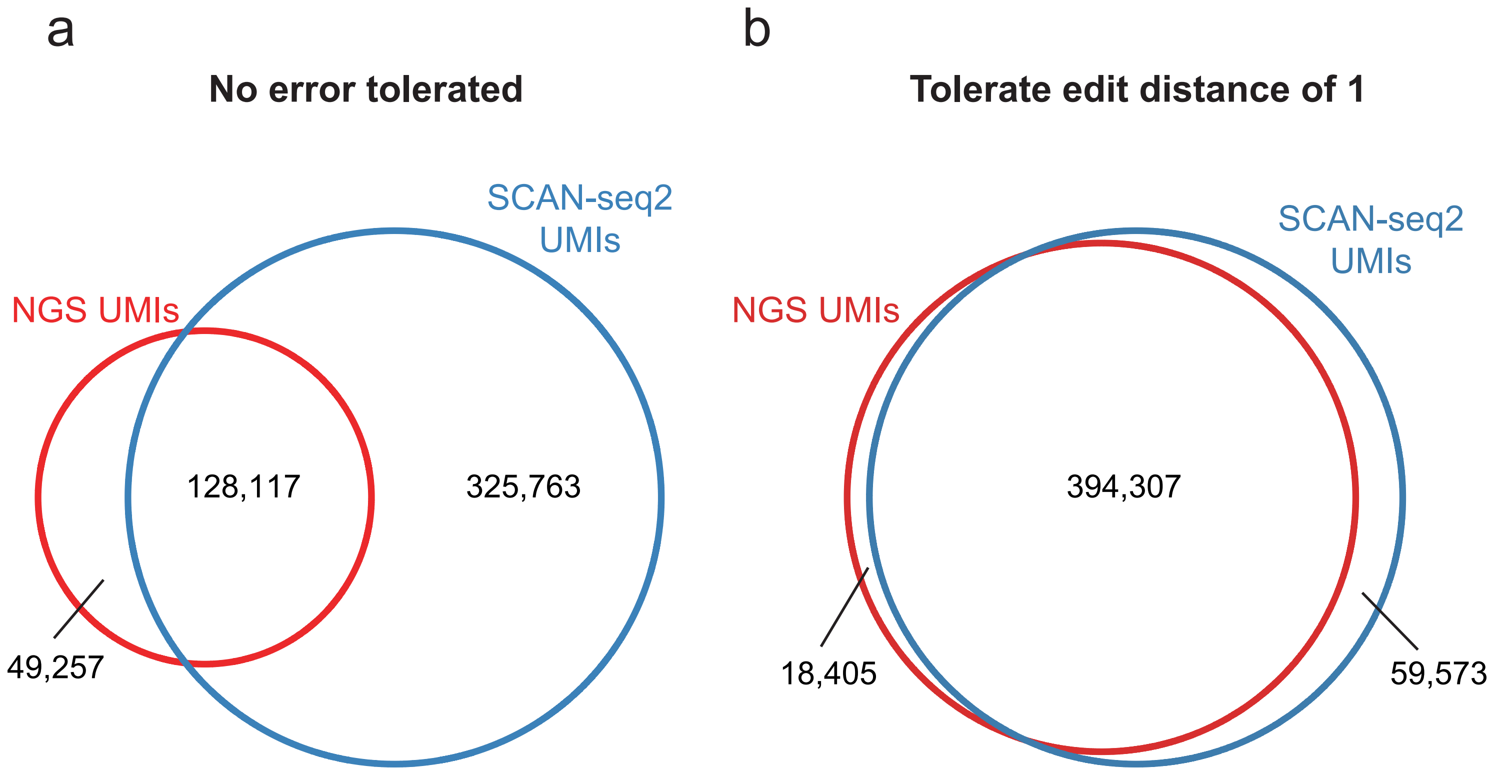

Supplementary Fig. S6

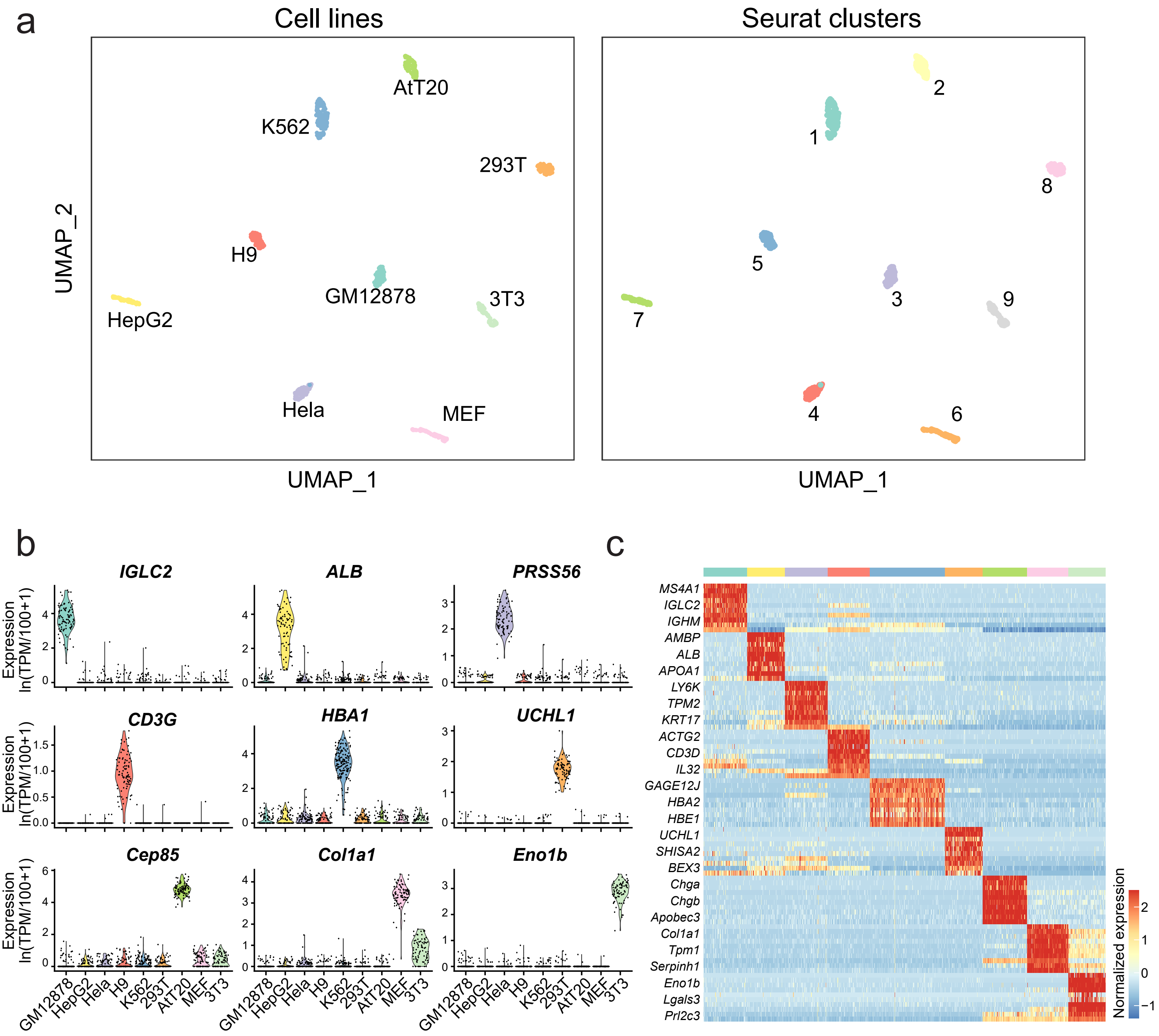

Supplementary Fig. S7

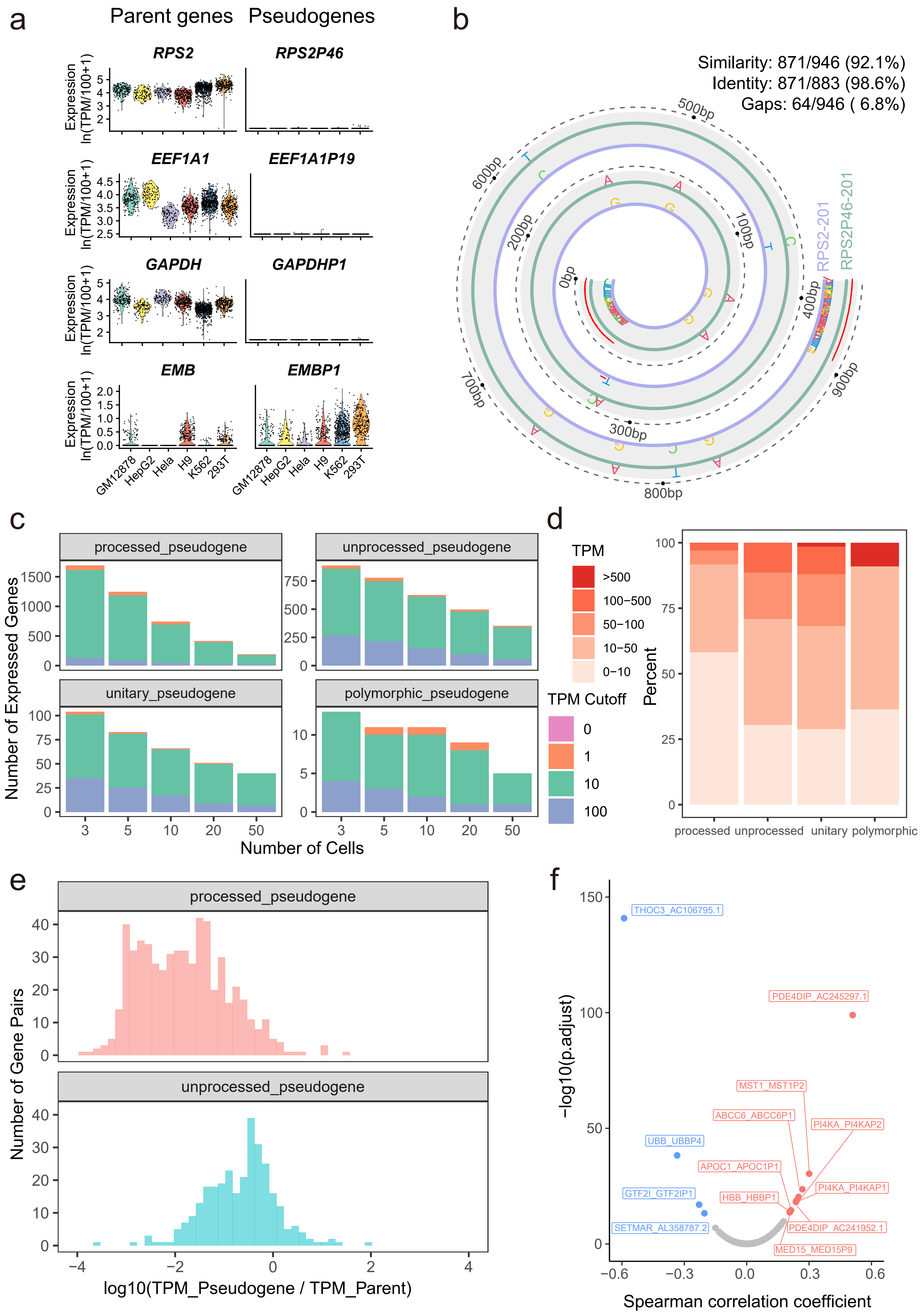

Supplementary Fig. S8

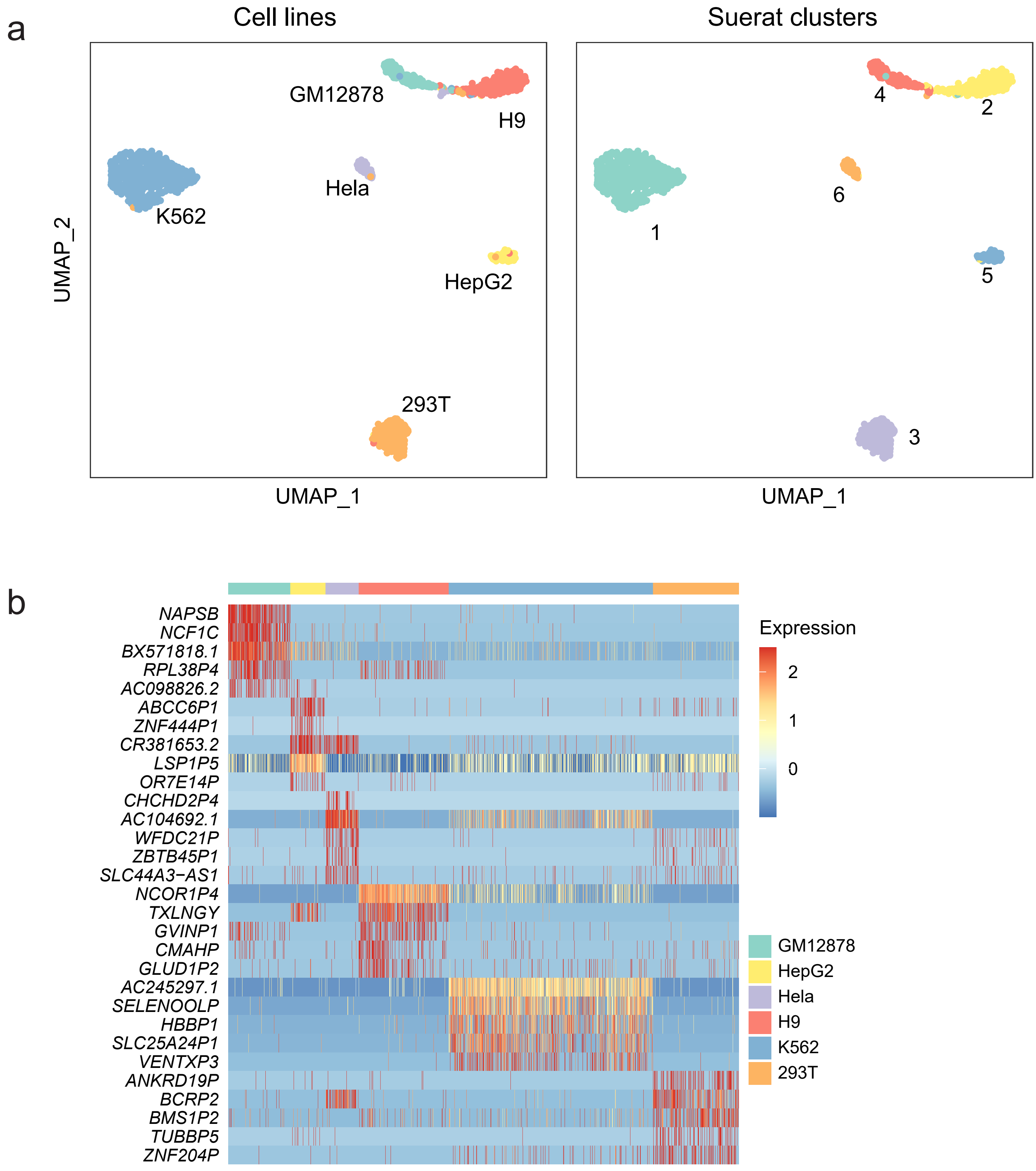

Supplementary Fig. S9

a

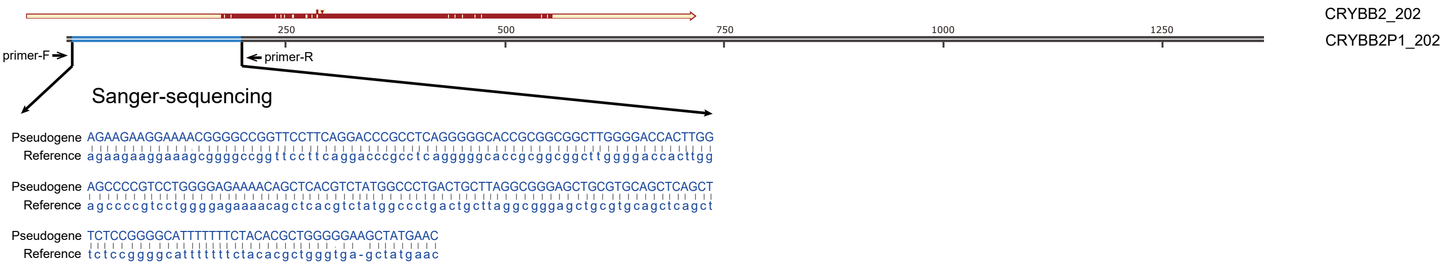

b

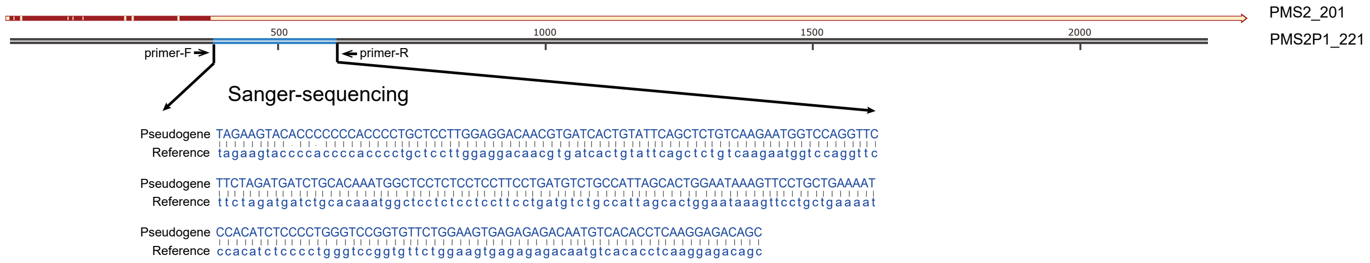

c

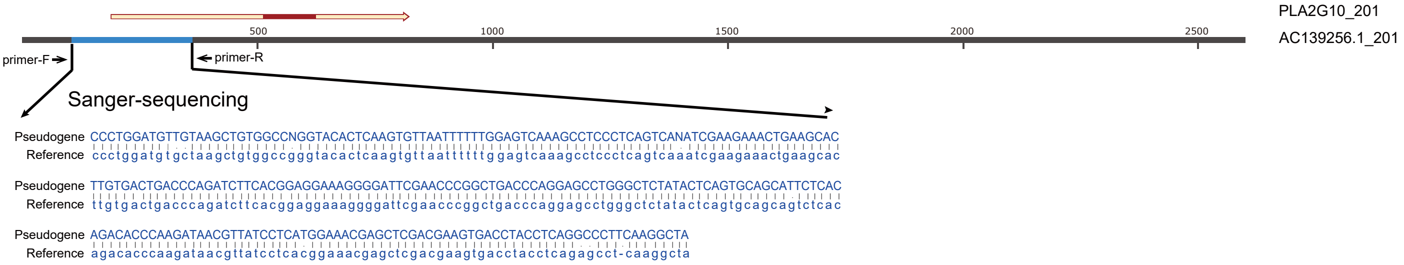

Supplementary Fig. S10

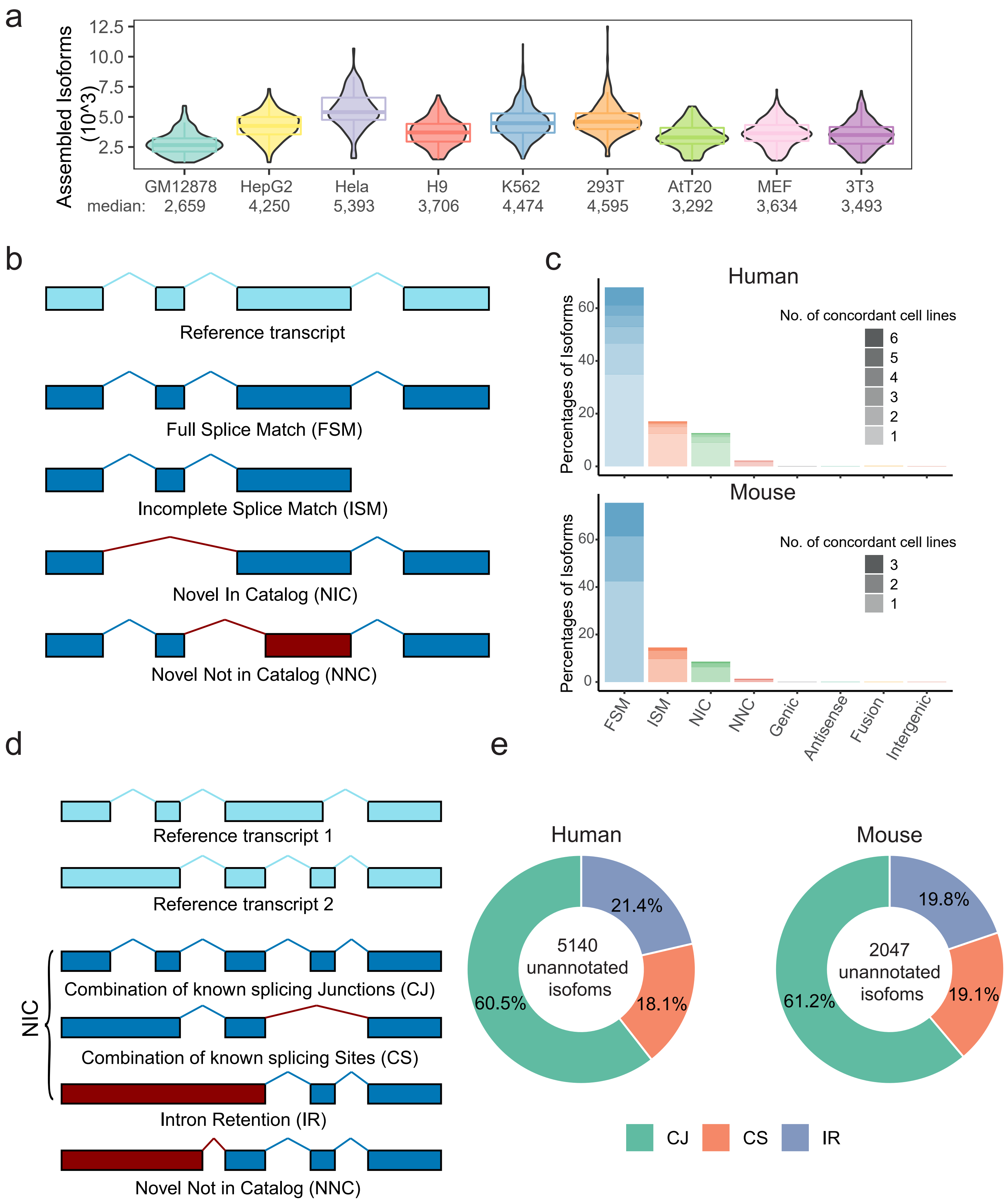

Supplementary Fig. S11

a

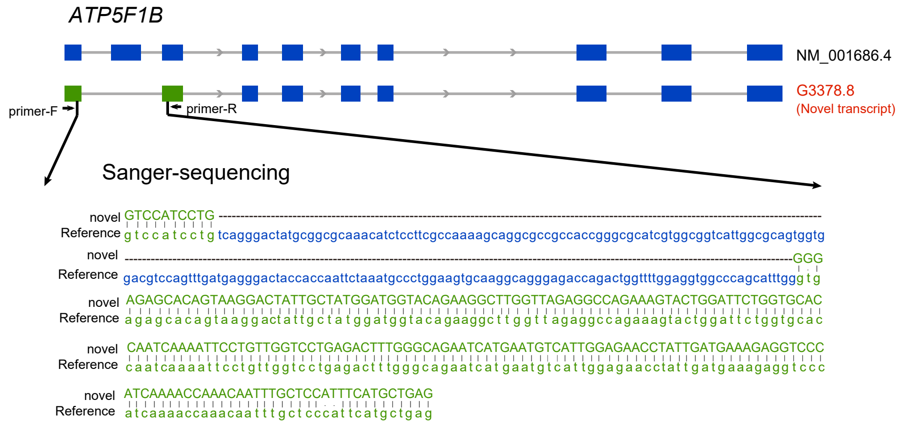

b

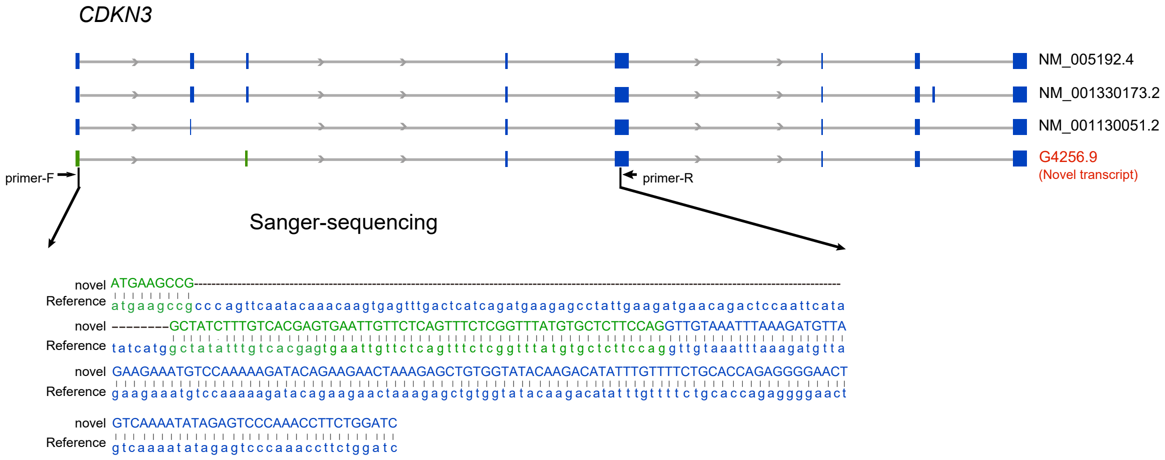

c

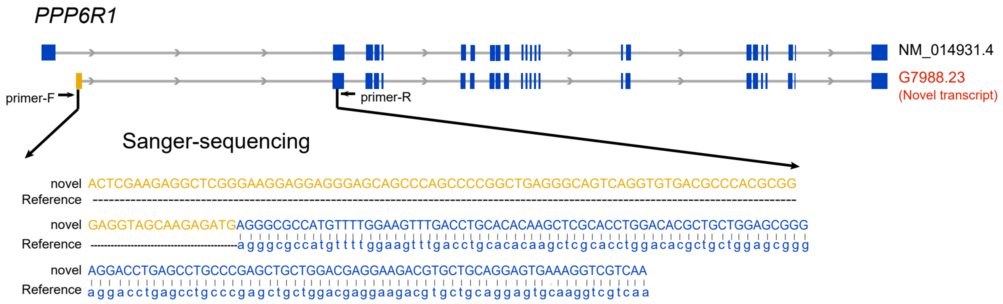

Supplementary Fig. S12

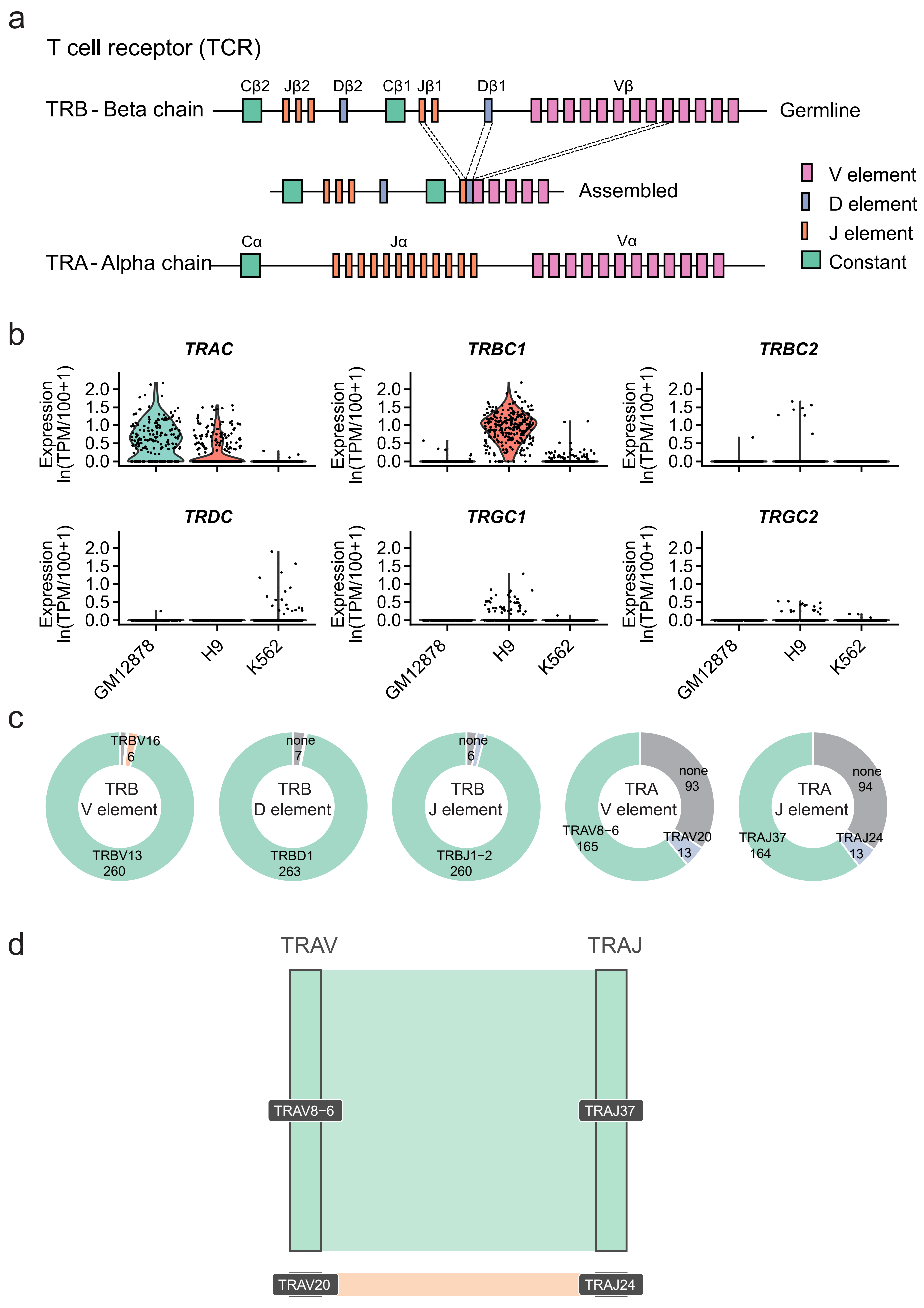

Supplementary Fig. S13

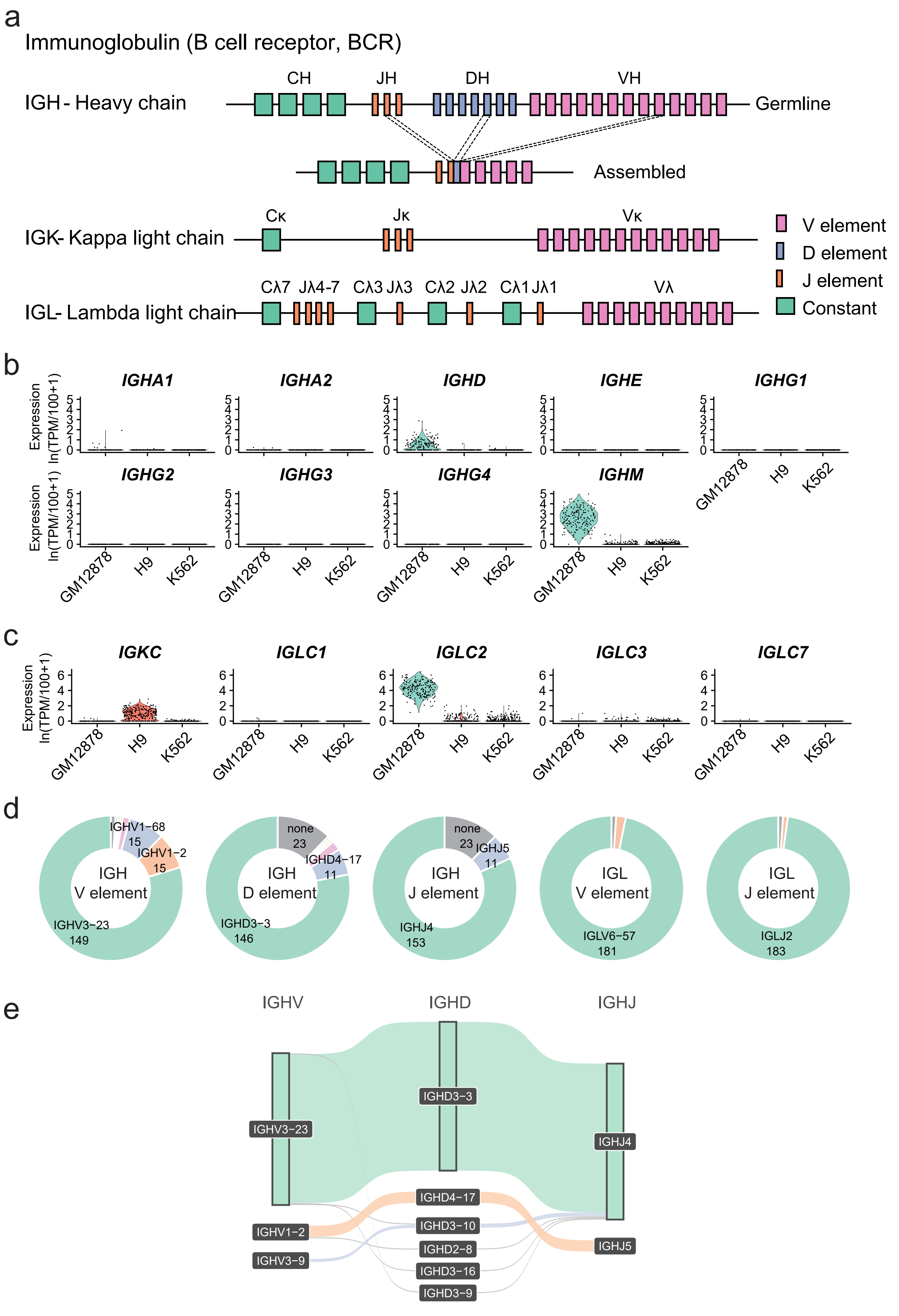

Supplementary Fig. S14

a

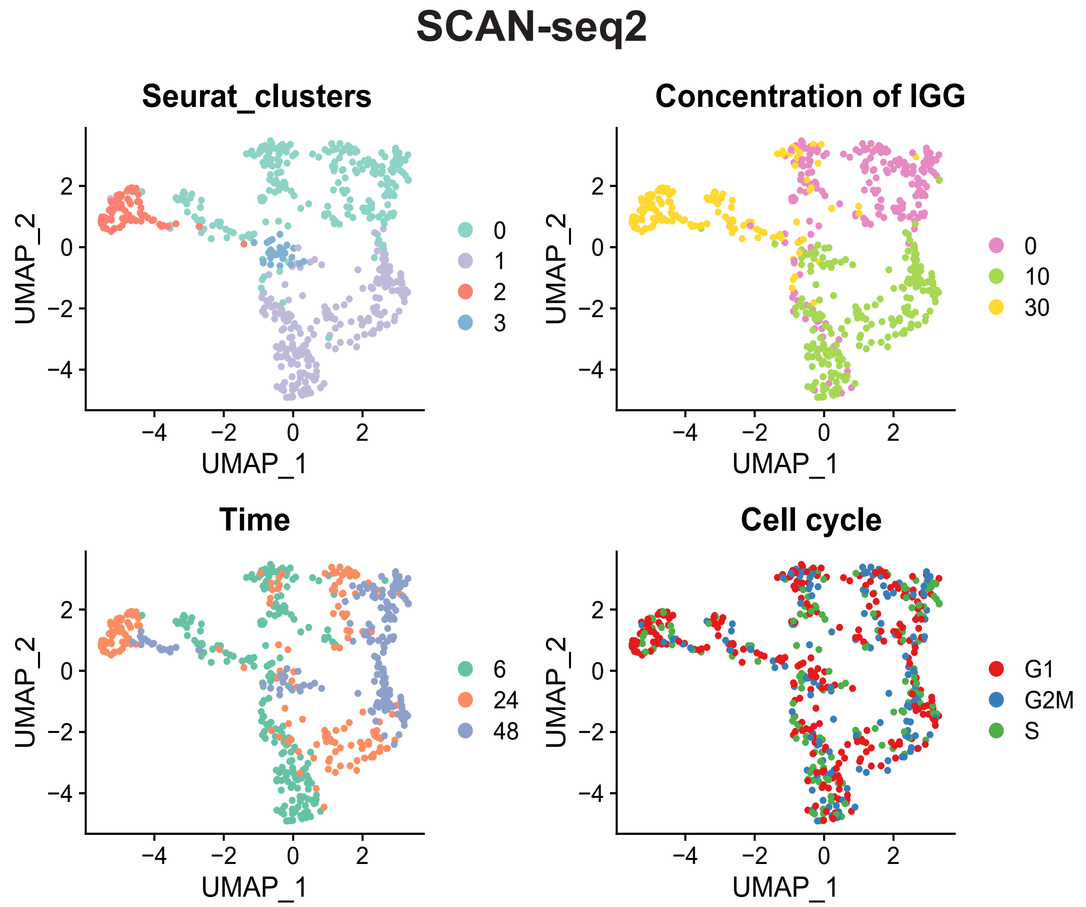

b

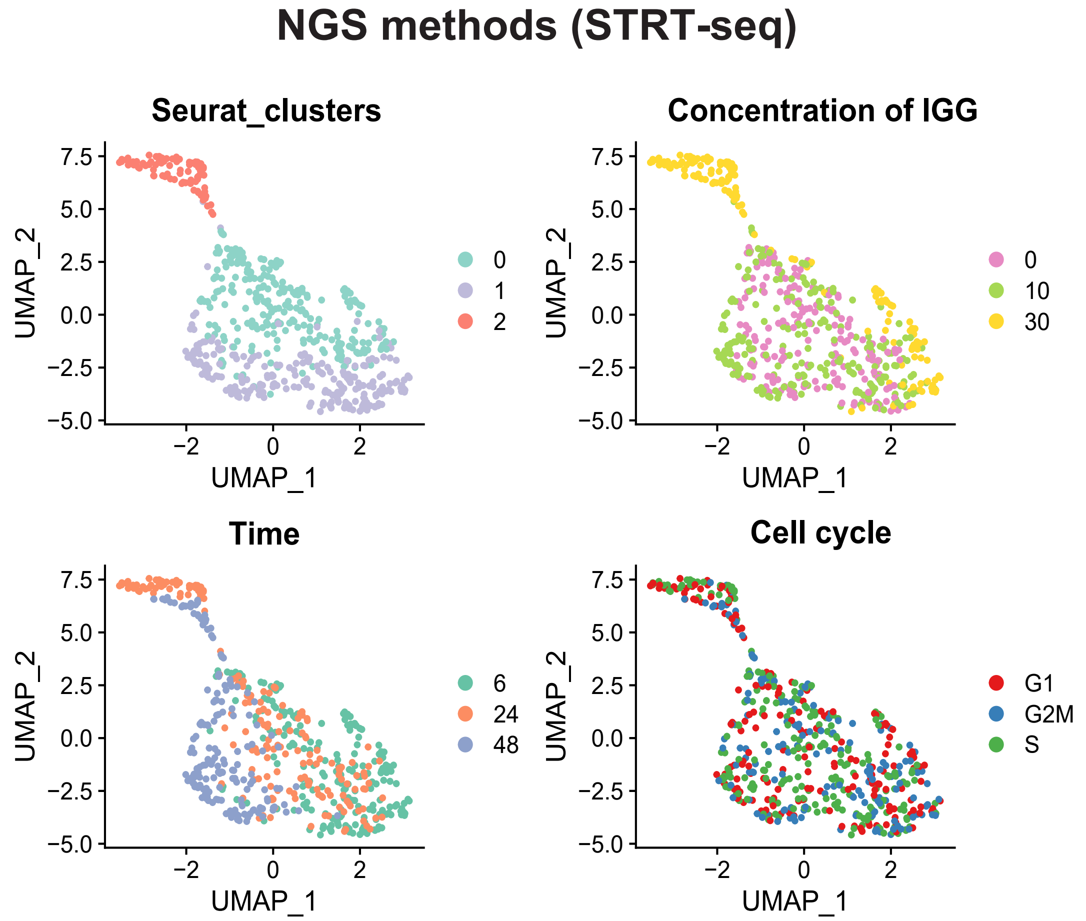

Supplementary Fig. S15

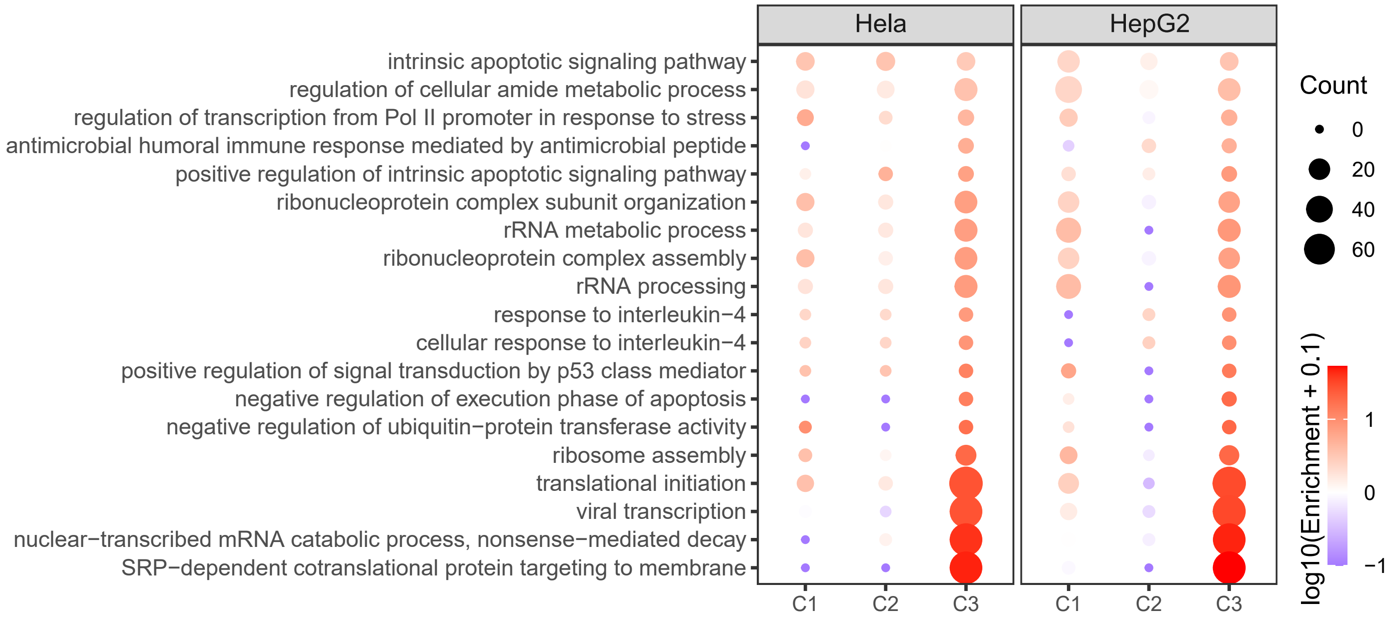

Supplementary Fig. S16

a

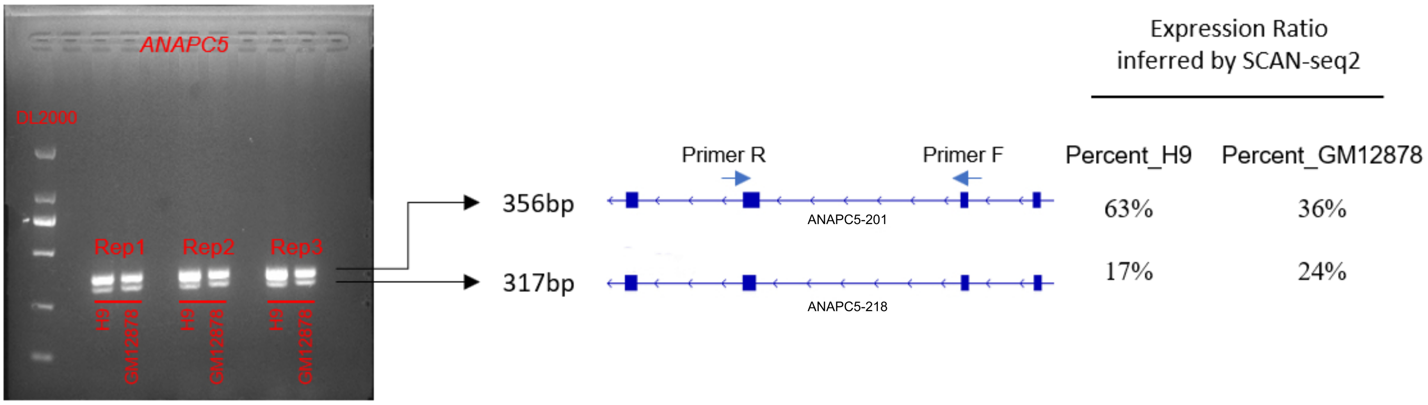

b

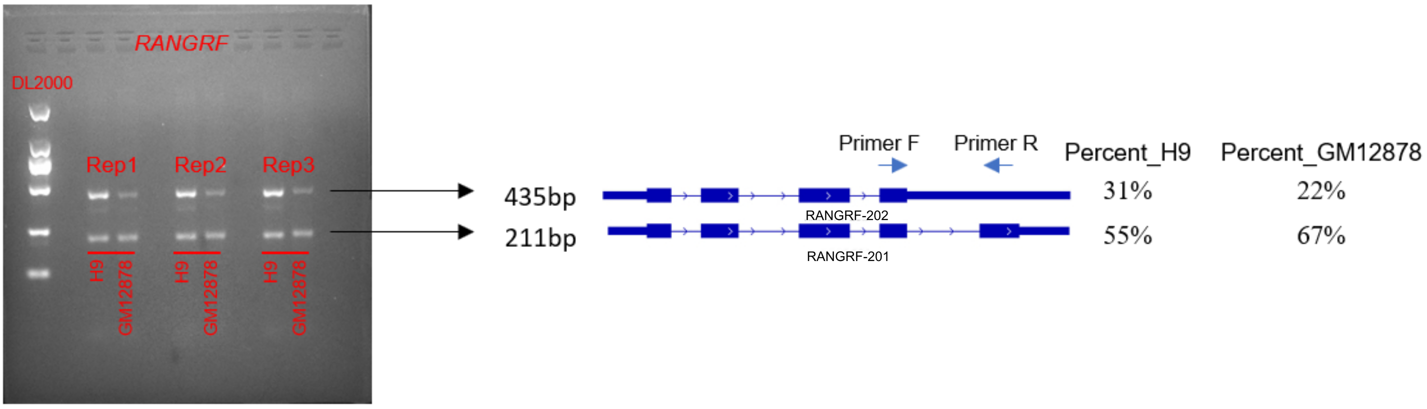

c

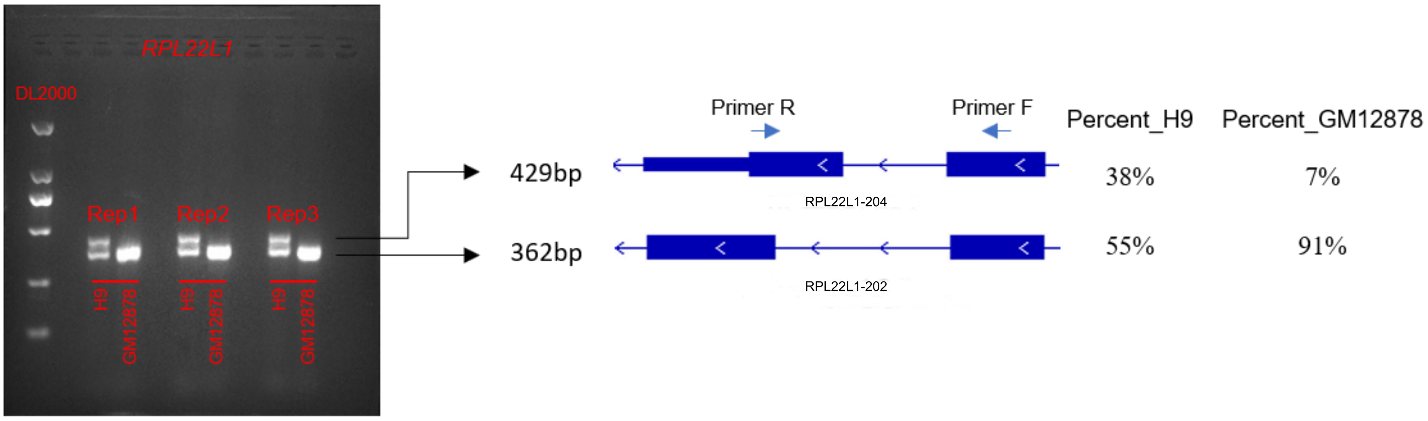

d

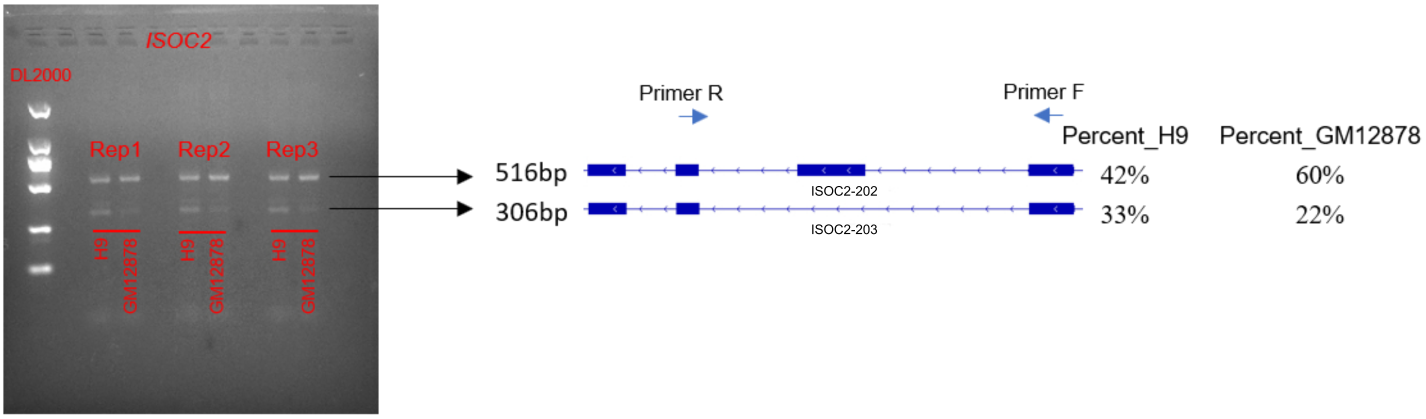

High-throughput and high-sensitivity full-length single-cell RNA-seq analysis on third-generation sequencing platform

Yuhan Liao[1,5], Zhenyu Liu[1,5], Yu Zhang[1,5], Ping Lu[1], Lu Wen[1] & Fuchou Tang[1,2,3,4*]

Materials and Methods

Experimental design

We set five groups to evaluate stability and reliability of SCAN-seq2. Two groups of 960 cells were from 9 cell lines (K562, HepG2, Hela, 293T, H9, GM12878 for human and MEF, 3T3, AtT20 for mouse). One of them was sorting individual cells from each cell line into 96-well plates with known identity for each cell (Library 9CL). The other were first mixing these nine cell lines together and then sorting individual cells into 96-well plates (Library 9CL-mix, parallel group #1 to #3). One group of 96 cells (Library UMI-100) and one group of 192 cells (Library UMI-200) were both derived from human K562 cell line and mouse 3T3 cell line. The last group was from 4 cell lines (human K562 & 293T cells and mouse MEF & 3T3 cells), and we mixed every 16 human cells and 16 mouse cells together after reverse transcription step and used the same 5' barcode primer to amplify them (Library 4CL).

Cell culture

K562 cells were maintained in RPMI 1640 medium, supplemented with 10% fetal bovine serum (FBS), 1% penicillin-streptomycin and 1% L-glutamine. GM12878 and H9 cells were both maintained in RPMI 1640 medium, supplemented with 10% FBS and 1% penicillin-streptomycin. HEK293T and MEF cells were both maintained in DMEM medium, supplemented with 10% FBS, 1% penicillin-streptomycin and 1% L-glutamine. Hela, HepG2, and 3T3 cells were maintained in DMEM medium, supplemented with 10% FBS, 1% penicillin-streptomycin. AtT20 cells were maintained in F12 medium, supplemented 15% horse serum (HS) and 2.5% FBS. All cell culture reagents were purchased from Gibco.

Single cell isolation

AtT20 cells were collected and washed with DPBS once, centrifuged at 300 rcf for 5 minutes, then resuspended with 1 mL medium. Same for K562 cells, H9 cells and GM12878 cells. For other 5 cells lines, cells were washed with DPBS, then digested with 0.05% trypsin at 37°C for 1-2 minutes. Cells were then centrifuged at 300 rcf for 5 minutes, resuspended with 1 mL medium. Followed by staining with 7-AAD, live single cells were sorted into individual wells of 96-well plates by FACS.

SCAN-seq2 single cell amplification

Cells from each line were sorted into 96-well plates containing lysis buffer. The lysis buffer comprised 2U RNase Inhibitor (Takara, Cat. 2313B), 0.475% Triton X-100 (Sigma-Aldrich, Cat. X100), 2.5 mM dNTP mixture (Thermo, Cat. R0193), 0.75 μM RT primer (TCAGACGTGTGCTCTTCCGATC-XXXXXXXXXXXXXXXXXXXXXXXX-N8-T25, with X representing the nucleotide of cell-specific barcode, N8 representing unique molecular identity), and 0.025% ERCC spike-in. Plates were thoroughly vortexed for 60 seconds and incubated at 72˚C for 10 minutes, so that the linearized RNA molecules were released. Then they were immediately transferred on ice. Next, we added 2.85 μL RT mixture into each well, which consisted of 10U Maxima H-minus RT enzyme (Thermo, EP0752), 2.5U RNase Inhibitor, 40 mM DTT, 12.5 mM MgCl2 (Sigma-Aldrich, Cat. 63020), 150 mM NaCl, 125 mM Tris-HCl pH8.3, 25% PEG 4000, 5 mM GTP, and 10 μM TSO primer (5' biotin-AAGCAGTGGTATCAACGCAGAGTACATrGrG+G, with rG representing riboguanosines and +G representing the locked nucleic acid (LNA)-modified guanosine). The RT reaction was carried out at 42˚C for 90 minutes, 11 cycles for 50˚C for 2 minutes,

42°C for 2 minutes, and 85°C for 15 minutes to deactivate the enzyme. After that, plates were spun down. We pooled mRNA-cDNA hybrid strands of every 32 cells with different 3' barcodes together and purified with 0.8X Ampure XP beads (Beckman, Cat. A63882) once. PCR mixture that included 2× KAPA HiFi Hot-Start Ready Mix, 266 nM 3' P2 primer (GTGACTGGAGTTCAGACGTGTGCTCTTCCGATC), 266 nM IS PCR oligo (ATGC-XXXXXXXXXXXXXXXXXXXXXXXX-AAGCAGTGGTATCAACGCAGAGT, with X representing the nucleotide of cell-specific barcode) was added into each tube. The amplification was performed by the following program: 4 cycles at 98°C for 20 seconds, 62°C for 30 seconds, and 72°C for 5 minutes, followed by 15 cycles at 98°C for 20 seconds, 67°C for 15 seconds, and 72°C for 5 minutes, with a final cycle at 72°C for 5 minutes. Then, we pooled the cDNAs with different 5' barcodes together and purified twice with 0.6X and 0.8X Ampure XP beads, respectively. And we quantified cDNA products with Equalbit® dsDNA HS Assay Kit (Vazyme, Cat. EQ111-01/02). Up to 1 μg cDNA products were used for further library construction.

SCAN-seq2 library preparation and sequencing

We constructed the library for Nanopore sequencing with Ligation Sequencing Kit 1D (ONT, Cat. SQK-LSK109). Briefly, the cDNA fragments were end-repaired and added dA-tailed using the Ultra II End Prep module (NEB, Cat. E7546). Then 1D adapters were tethered to above products by Quick Ligation Module (NEB, Cat. E6056). After that, each cDNA library was loaded into one R9.4 chip and sequenced on PromethION 48.

IGG treatment

Isoginkgetin (IGG) (MCE, Cat. HY-N2117) was dissolved in DMSO and stored in -80°C. Hela and HepG2 cells were pre-cultured to achieve sufficient amount. Then cells were equally divided into 96-well plates and each well contained 3,000 cells and 100 μL medium. We set three IGG concentration gradients (0 μM, 10 μM, and 30 μM) and added them to the culture medium correspondingly. Both cell lines had at least three parallel groups for each gradient. Then we digested cells at 6 h, 24 h, and 48 h after dosing treatment, respectively. Living cells were sorted by FACS after 7-AAD staining, and 2,304 cells were collected in total.

SCAN-seq2 data processing

Base calling was performed on the electric signals of nanopore sequencing to generate fastq files using Guppy (v4.0.1). Single-cell barcodes were extracted from 150 bp on both ends of the reads by nanoplexer (v0.1.2, https://github.com/hanyue36/nanoplexer). Reads with low quality (q < 7) and short length (< 100 bp) were filtered out using NanoFilt (v2.7.1) [1]. Pychopper (v2.5.0, https://github.com/nanoporetech/pychopper) was utilized to trim adaptors, identify and orient full-length cDNA sequence. UMIs were extracted from 3' end of reads and added to header by UMI-tools (v1.0.1) [2] extract command. Poly-A sequences were trimmed using cutadapt (v3.2) [3] with parameters '-a "A{10}" -e 0.2'. Clean reads were mapped to a merged reference transcriptome of human (GRCh38.p13) and mouse (GRCm38.p6) cDNAs from Ensembl release 101 using minimap2 (v 2.17-r941) [4]. PCR duplications were filtered based on UMI sequence and mapping position using dedup command of UMI-tools (v1.0.1) [2] with parameters "--method=directional --edit-distance-threshold=1 --per-gene --per-contig --buffer-whole-contig". Expression levels of each transcript and each gene were quantified using Salmon (v1.3.0) [5] in alignment-based mode. Transcript per million (TPM) of each gene and transcript was calculated as UMI count per million unique UMIs.

Pseudogene analysis

In the human and mouse genomes, a number of pseudogenes involving in complex gene regulatory networks and with potential as cancer biomarkers were identified. There are four major types of pseudogenes in human genome - the processed (~10,000), unprocessed (~3,500), unitary (~200) and polymorphic (~50) pseudogenes with different mechanisms of origin [6]. We identified pseudogenes based on Ensembl annotations. Genes with following biotypes were defined as pseudogenes in this research: processed pseudogene, unprocessed pseudogene, unitary pseudogene, polymorphic pseudogene. Other pseudogenes, including rRNA pseudogenes and pseudogenes of immunoglobin and T cell receptor were not included. For processed pseudogene and unprocessed pseudogene, corresponding parent genes were also identified based on Ensembl gene annotation.

The expression level of pseudogenes was defined as the average TPM of top 100 cells with highest expression level of this gene. Correlation between pseudogene and parent gene was evaluated by Spearmans rank -order correlation. The absolute value of correlation coefficient larger than 0.2 was considered significant.

Dimensionality reduction and clustering

Further analysis based on expression matrix was performed with Seurat package (v4.0.3) [7]. Cells with less than 2000 genes detected or more than 15% mitochondrial UMIs were discarded. Top 2,000 highly variable genes were selected with Seurat FindVariableFeatures function. Principle component analysis (PCA) was then performed on highly variable genes. Unsupervised clustering of cells was performed using original Louvain algorithm of Seurat FindClusters function. UMAP embedding was calculated to visualized cluster and cell type information.

Differential gene expression (DGE) and differential transcript usage (DTU) analysis

Differential gene expression analysis was performed using Wilcoxon rank sum test on log2(transcript per million) value. Genes with absolute log2-transformed fold change of >1, and an adjusted P value of $P < 0.05$ were considered as differentially expressed.

Differential transcript usage analysis was performed with R package DTUrtle (v1.0.2) [8]. First, transcripts with less than 5 UMI support or detected in less than 25 cells were removed to reduce multiple testing. The Dirichlet-multinomial model was used to estimate the precision parameter. Next, a group-wise maximum likelihood estimation of transcript proportions was calculated. A likelihood ratio test for transcript proportions was used to identify DTU. Genes with false discovery rate (FDR) < 0.05 were considered significant.

Transcriptome assembly

Unique reads after deduplication were mapped to reference genome of human (GRCh38.p13) or mouse (GRCm38.p6) from Ensembl release 101 using minimap2 (v 2.17-r941) [4]. Reads with MAPQ < 30 were discarded. We performed transcriptome assembly for each single cell using StringTie (v2.1.7) [9] in long reads processing mode. Gene annotation gtf files from Ensembl were used to guide the assembly process. Single-cell assemblies were classified using the sqanti3_qc.py script of SQANTI3 (v3.4.1) [10] with the parameters "--skipORF --report pdf" and then filtered using the sqanti3_RulesFilter.py script of SQANTI3 (v3.4.1) with default parameters.

Single-cell transcriptome assemblies were integrated into merged assembly in a hierarchical manner using the merge subcommand of TAMA (v0.0) [11]. Briefly, single-cell assemblies were first merged into 9 cell-line assemblies with the parameter "-a 100 -m 20 -z 50". Only transcript

supported by more than 5 cells were retained in cell line assemblies. Next, 6 cell-line assemblies for human and 3 cell-line assemblies for mouse were merged respectively with the parameter "-a 10 -m 10 -z 10". The merged assemblies were then compared with Ensembl gene annotation (release 101) and filtered using SQANTI3 (v3.4.1) with identical parameters as used for single-cell assemblies.

Consensus and polishing of TCR and immunoglobin (BCR) sequences

In each GM12878 or H9 cell, reads mapped to immunoglobin or T cell receptor gene locus (IGH: chr14:105,586,437-106,879,844; IGL: chr22:22,026,076-22,922,913; TRA: chr14:21,621,904-22,552,132; TRB: chr7:142,299,011-142,813,287) were extracted. For each single cell, extracted reads were initially clustered using usearch (v.11.0.667) [12] -cluster_fast -id 0.75 -sizeout -centroids. The centroid read of the largest group was selected as representative sequence and used as the template for 4 rounds of polishing using all reads from the same cluster with minimap2 (v 2.17-r941) [4] -x map-ont followed by racon (v1.5.0) [13] -w 200 -m 8 -x -6 -g -8 -q 7. The racon-polished sequence was further corrected using all reads of the same cluster with Medaka (https://github.com/nanoporetech/medaka) consensus -m r941_min_high_g360. The medaka-corrected sequences were regarded as the TCR/immunoglobin transcripts and were utilized for the identification of V(D)J recombination. This pipeline was reported to produce sequence of ~99.995% accuracy at 25X coverage during amplicon sequencing [14].

Identify V(D)J recombination for TCR and immunoglobin (BCR)

Genes encoding variable regions of B- and T- lymphocyte antigen receptors are assembled by recombination of variable (V), diversity (D), and joining (J) gene segments [15-16]. The V(D)J elements in the corrected TCR/immunoglobin transcripts were identified with Igblast (v 1.17.1) [17] with parameters "-organism human -show_translation -outfmt 19" for immunoglobin and an extra parameter "-ig_seqtype TCR" for TCR. Annotation for human VDJ elements were downloaded from the international immunogenetics information system (IGMT). Hits with the smallest E value were retained for each cell.

Subclones of GM12878 and H9 cells were identified based on the V(D)J combination of each cell. Briefly, GM12878 cells with the same VDJ elements for immunoglobin heavy chain and same VJ elements for light chain were considered as the same subclone. H9 cells with the same VDJ elements for β chain and same VJ elements for α chain were considered as the same subclone. Subclones with more than 5 cells were considered solid and retained for further analysis.

Availability of data and materials

All relevant data are available from the Gene Expression Omnibus (GEO) database (accession number: GSE203561).

References

[1] De Coster, W. et al. NanoPack: visualizing and processing long-read sequencing data. *Bioinformatics* **34**, 2666-2669 (2018).

[2] Smith, T., Heger, A. & Sudbery, I. UMI-tools: modeling sequencing errors in Unique Molecular Identifiers to improve quantification accuracy. *Genome Research* **27**, 491-499 (2017).

[3] Martin M. Cutadapt removes adapter sequences from high-throughput sequencing reads. *EMBnet.journal* **17**, 10-12 (2011)

[4] Li, H. Minimap2: pairwise alignment for nucleotide sequences. *Bioinformatics* **34**, 3094-3100 (2018).

[5] Patro, R., Duggal, G., Love, M. I., Irizarry, R. A. & Kingsford, C. Salmon provides fast and bias-aware quantification of transcript expression. *Nature Methods* **14**, 417-419 (2017).

[6] Qian, S. H., Chen, L., Xiong, Y.-L. & Chen, Z.-X. Evolution and function of developmentally dynamic pseudogenes in mammals. *Genome Biology* **23** (2022).

[7] Hao, Y. et al. Integrated analysis of multimodal single-cell data. *Cell* **184**, 3573-3587.e3529 (2021).

[8] Tekath, T., Dugas, M. & Boeva, V. Differential transcript usage analysis of bulk and single-cell RNA-seq data with DTUrtle. *Bioinformatics* **37**, 3781-3787 (2021).

[9] Kovaka, S. et al. Transcriptome assembly from long-read RNA-seq alignments with StringTie2. *Genome Biology* **20** (2019).

[10] Tardaguila, M. et al. SQANTI: extensive characterization of long-read transcript sequences for quality control in full-length transcriptome identification and quantification. *Genome Research* **28**, 396-411 (2018).

[11] Kuo, R. I. et al. Illuminating the dark side of the human transcriptome with long read transcript sequencing. *BMC Genomics* **21** (2020).

[12] Edgar, R. C. Search and clustering orders of magnitude faster than BLAST. *Bioinformatics* **26**, 2460-2461 (2010).

[13] Vaser, R., Sović, I., Nagarajan, N. & Šikić, M. Fast and accurate de novo genome assembly from long uncorrected reads. *Genome Research* **27**, 737-746 (2017).

[14] Karst, S. M. et al. High-accuracy long-read amplicon sequences using unique molecular identifiers with Nanopore or PacBio sequencing. *Nature Methods* **18**, 165-169 (2021).

[15] Schatz, D. G. & Ji, Y. Recombination centres and the orchestration of V(D)J recombination. *Nature Reviews Immunology* **11**, 251-263 (2011).

[16] De Simone, M., Rossetti, G. & Pagani, M. Single Cell T Cell Receptor Sequencing: Techniques and Future Challenges. *Frontiers in Immunology* **9** (2018).

[17] Ye, J., Ma, N., Madden, T. L. & Ostell, J. M. IgBLAST: an immunoglobulin variable domain sequence analysis tool. *Nucleic Acids Research* **41**, W34-W40 (2013).

Figure legend

Fig. 1 SCAN-seq2 technical performances and analysis of Isoginkgetin (IGG) responses in cell lines. a. Schematic diagram of SCAN-seq2 library construction. N different single cells are labeled with 3' barcode during reverse transcription and pooled into the same tube for PCR amplification. M different tubes are pooled together and sequenced with Nanopore platform, allowing parallel sequencing of N×M cells. b. Number of detected genes (top) and isoforms (bottom) in 852 cells from library 9CL. Median values are labeled under each cell line. c. Pearson correlation of ERCC concentration and sequenced UMIs in each library. d. Correlation between gene expression quantification of SCAN-seq2 and Smart-seq3 in 293T cells. Single cells are aggregated into pseudo-bulk for comparison. e-f. UMAP embeddings of Hela (e) and HepG2 (f) cells after IGG treatment at different concentration and time. Cells are colored by unsupervised clustering results (top left), cell cycle phase (top right), IGG concentration (bottom left) and time of treatment (bottom right). IGG-responsive clusters are highlighted in red circles. g. Venn diagram showing the overlap in upregulated differentially expressed genes (DEGs) and differential transcript usage (DTU) in IGG-responsive cluster of Hela and HepG2 cells. h. Venn diagram of DEGs and genes with significant DTU in IGG-responsive cluster of Hela cells. i. Fraction of each subcategory for NIC transcripts in different clusters. P values are calculated by two-tailed Wilcoxon rank-sum test. *: $p < 0.05$, **: $p < 0.01$, ***: $p < 0.001$. j. Examples of genes with significant differential transcript usage in IGG-responsive cluster. Exons with different usage are highlighted in red.

Supplementary Fig. S1 Flowchart of SCAN-seq2 data processing. a. Demultiplexing, trimming, filtering, and deduplication of raw Nanopore reads. b. Isoform expression quantification and reference-guided transcriptome assembly. Software utilized in each step is indicated next to the lines. The nodes are colored by the scale of the data, either single-cell level (green), cell line level (red), or all cells merged (purple). The number of reads, genes, and transcripts is also labeled in each node. For single cell level analysis, the number is calculated as the average value of all cells. The file format of each node is labeled in brackets.

Supplementary Fig. S2 Statistics of SCAN-seq2 reads after each processing step. a. Boxplot of reads statistics after demultiplexing, quality control, and deduplication in every single cell. Mean_read_quality (Q-score) b. Circle plot showing number of reads and total bases retained after each processing step. The total yield of a single PromethION is defined as a whole circle.

Supplementary Fig. S3 Overview of SCAN-seq2. a. Structure of SCAN-Seq2 library. Barcodes are introduced to both ends of cDNA for massively parallel analysis of thousands of single cells. b.

Donut chart showing the distribution of sequenced reads from library 9CL. 75.9% of reads have complete library structure and can be used for downstream analysis. c. Fragment analysis (FA) of library 9CL. d. Histogram indicating the length of cDNA sequence after adaptor trimming. e. Mapping rate of reads from each cell line. The median values are labeled below. f. Barnyard plot of 710 cells from library 4CL. Dotted line indicates specificity of 90%. Red dots indicate human-specific barcodes. Blue dots indicate mouse-specific barcodes. One barcode associated with both human and mouse transcript (Gray). g. Saturation analysis of SCAN-seq2. Number of detected genes and isoforms reach plateau at 400,000 reads per cell. h. Cell-to-cell correlation of SCAN-seq2 gene quantification in 293T cells, comparing with Smart-seq3. i. Expression level of *PTPRC* gene (top left) and its protein-coding isoforms in 3 immune cell lines. GM12878 cells mainly utilize PTPRC-209 while K562 cells mainly utilize PTPRC-201. j. Alternative splicing at exon 4 (A), 5 (B), 6 (C) of *PTPRC* gene. PTPRC-201 uses none of these three exons, encoding CD45 RO. PTPRC-209 uses all three exons, thus encoding CD45 RABC.

Supplementary Fig. S4 Evaluation of sequencing errors in 24-bp barcode sequences. Errors of Nanopore sequencing, including mismatched (top), indel (middle), and overall errors (bottom) were counted in each demultiplexed barcode. a. 5 prime barcodes. b. 3 prime barcodes.

Supplementary Fig. S5 Comparison of SCAN-seq2 UMIs with those in NGS methods. UMIs of ERCC reads identified by SCAN-seq2 are compared to those identified by Illumina sequencing of the same library (but the original SCAN-seq2 sequencing library has been fragmented into about 300bp fragments and the fragments containing 3' ends of the original library are captured by biotin-streptavidin affinity strategy and further ligated into NGS sequencing adaptor pairs). a. No sequence error is tolerated. b. Tolerated sequence error at an edit distance of 1.

Supplementary Fig. S6 SCAN-seq2 analysis of 9 different cell lines. a. UMAP embedding of library 9CL. Cells are colored by cell line (left) and unsupervised clusters (right). b. Violin plot showing selected markers of each cell line. c. Heatmap of cell-line specific markers. For each cell line, 5 genes with highest fold change against other cells are included.

Supplementary Fig. S7 Systematic evaluation of pseudogene expression in human cell lines using SCAN-seq2. a. Expression measurements in SCAN-seq2 of selected pseudogenes and corresponding parent genes. 4 gene pairs with identity 95% are selected. b. Spiral chart showing pairwise sequence alignment of RPS2-201 (purple) and RPS2P46-201 (green) transcript. Gaps are

highlighted in red. Mismatched bases and gap sequence are labeled by corresponding nucleobases. c. Number of expressed genes under different TPM cutoff and number of supported cells. Pseudogenes are grouped by gene type from Ensembl annotation. d. Fraction of expressed pseudogenes with different expression level. TPM of each gene is calculated as the average expression level of top 100 cells with highest expression level. e. Comparison of expression levels of pseudogenes and corresponding protein-coding parent genes. Pseudogenes generally possess significantly lower expression level comparing with parent gene. f. Correlation analysis of pseudogene expression and parent gene expression in 6 human cell lines. Pairs with absolute value of spearman correlation coefficient > 0.25 are considered significant. Significant pairs are labeled with the symbol of parent genes and pseudogene. Positively correlated pairs are colored in red. Negatively correlated pairs are colored in blue.

Supplementary Fig. S8 Unsupervised clustering on pseudogene expression distinguishes different cell lines. a. UMAP embedding of 6 human cell line. Reductions are calculated on pseudogenes only. Cells are colored by cell line (left) and unsupervised clusters (right). b. Heatmap of cell-line specific pseudogene markers. For each cell line, 5 pseudogenes with highest fold change against other cells are included.

Supplementary Fig. S9 Examples of pseudogenes identified in H9 and GM12878 cells. Schematic displays 3 pseudogene-parent gene pairs (pseudogene *CRYBB2P1* and parent gene *CRYBB2*, pseudogene *PMS2P1* and parent gene *PMS2*, pseudogene *AC139256.1* and parent gene *PLA2G10*) (a-c). The red parts indicate regions of identical sequences between the pseudogene and corresponding parent gene. The blue parts are pseudogene-specific regions, which were amplified by RT-PCR followed by Sanger-sequencing. Details are showed in Supplementary Table. S8.

Supplementary Fig. S10 Transcriptome assembly using SCAN-seq2. a. Number of assembled isoforms in each single cell. The median values of each cell line are labeled below. b. Schematic diagram of isoform classification by comparing with gene annotations. Disagreements with annotations are highlighted in red. Full splice match (FSM) and incomplete splice match (ISM) indicate splicing events conforming to annotations. Novel in catalog (NIC) indicates combination of known splicing site and junctions. Novel not in catalog (NNC) indicates novel splicing sites. c. Fraction of each isoform classification in 6 human cell lines (top) and 3 mouse cell lines (bottom). Transparency of bars indicates number of concordant cell lines. Isoforms detected in all cell lines are colored with lowest transparency. d. Schematic diagram of unannotated transcript classification.

Disagreements with annotations are highlighted in red. CJ indicates unannotated combination of splicing junctions. CS utilizes known splicing sites to create unannotated junctions. IR indicates retained intron. NNC includes unannotated splicing sites. e. Donut chart demonstrating fraction of each NIC subcategories in 6 human cell lines (left) and 3 mouse cell lines (right).

Supplementary Fig. S11 Examples of unannotated transcripts identified in H9 and GM128278 cells. a. Transcript G3378.8 matches partially to *ATP5F1B* gene sequences, but lacks the second exon. b. *CDKN3* gene has three annotated transcripts whereas transcript G4256.9 lacks the second exon compared with the known three transcripts. c. Transcript G7988.23 shares a portion of sequences with *PPP6R1* gene, but uses an alternative transcription start site (TSS). Details are showed in Supplementary Table. S8.

Supplementary Fig. S12 T cell receptor (TCR) analysis of H9 cell line. a. Schematic diagram of V(D)J recombination in TCR beta chain (TRB) and alpha chain (TRA). In the assembled genome, only a single VDJ element is retained for expression. b. Expression of all TCR constant region genes in 3 immune cell lines. H9 cells mainly utilize *TRBC1* for beta chain. c. Identification of VDJ elements in TRB transcript and VJ in TRA transcript in each single cell. Elements with more than 5 cells are labeled. Cells with insufficient reads or no significant hits are labeled as none. d. Subclone analysis of H9 cell line. Two distinct clones with different TRAV and TRAJ elements are identified.

Supplementary Fig. S13 Immunoglobin (BCR) analysis of GM12878 cell line. a. Schematic diagram of V(D)J recombination in Immunoglobin heavy chain (IGH), kappa light chain (IGK) and lambda light chain (IGL). b. Expression of all immunoglobin heavy chain constant region genes in 3 immune cell lines. Transcripts of gene *IGHM* and *IGHD* are detected in GM12878 cells, indicating that most GM12878 cells express immunoglobulin M (IgM) whereas a small proportion of GM12878 cells express immunoglobulin D (IgD). c. Expression of all immunoglobin light chain constant region genes in 3 immune cell lines. GM12878 cells only utilize *IGLC2* from lambda light chain locus for light chain construction. d. Identification of VDJ elements in IGH transcript and VJ in IGL transcript in each single cell. The D element of IGL is not detected, and VJ rearrangement is same in essentially all GM12878 cells. Elements with more than 5 cells are labeled. Cells with insufficient reads or no significant hits are labeled as none. E. Subclone analysis of GM12878 cell line. Three distinct clones with different VDJ elements are identified.

Supplementary Fig. S14 Comparison of clustering on IGG-treated Hela cells by SCAN-seq2

and NGS methods. UMAP embeddings of Hela cells after IGG treatment at different concentrations and times. scRNA-seq was performed with both SCAN-seq2 (a) and NGS-based STRT method (b). Cells are colored by unsupervised clustering results (top left), IGG concentration (top right), time of treatment (bottom left) and cell cycle phase (bottom right).

Supplementary Fig. S15 GO enrichment analysis in IGG-responsive cluster in 2 cell lines. Dots are colored by fold of enrichment and sized by the number of relevant genes.

Supplementary Fig. S16 Validation of DTU events between H9 and GM12878 cell lines. DTU events in 4 genes (*ANAPC5*, *RANGRF*, *RPL22L1*, *ISOC2*) are shown (a-d). For each DTU event, two different cDNA products from transcripts of different lengths from a specific gene are amplified by one pair of primers. The expression ratios inferred by SCAN-seq2 are listed on the right.

Acknowledgments

This work was supported by the Beijing Advanced Innovation Center for Genomics at Peking University. We thank members in the Tang laboratory for discussions. Thank Dr. Ping Lu in construction of bioinformatics analysis process.

Funding

The work was supported by the National Key R&D Program of China (2021ZD0200102, 2022YEF0203200 and 2018YFA0107601) and National Natural Science Foundation of China (32288102 and 31871457).

Author contributions

FT conceived the project. YL and YZ developed the protocol and contributed to cell culture, flow cytometry plus the cDNA sample preparation. ZL was in charge of the bioinformatic analysis. YL, YZ, ZL and FT wrote the manuscript with help from all authors.

Ethics declarations

Ethics approval and consent to participate

Not applicable.

Consent for publication

Not applicable.

Conflict of interest

The authors declare no competing interests.
